# Detection of stable QTLs for grain protein content in rice (*Oryza sativa* L.) employing high throughput phenotyping and genotyping platforms

**DOI:** 10.1038/s41598-019-39863-2

**Published:** 2019-03-01

**Authors:** Krishnendu Chattopadhyay, Lambodar Behera, Torit Baran Bagchi, Sushree Sibanee Sardar, Nutan Moharana, Niraja Rani Patra, Mridul Chakraborti, Avijit Das, Bishnu Charan Marndi, Ananta Sarkar, Umakanta Ngangkham, Koushik Chakraborty, Lotan Kumar Bose, Sutapa Sarkar, Soham Ray, Srigopal Sharma

**Affiliations:** 1 ICAR-National Rice Research Institute, Cuttack, India; 2 ICAR-National Institute of Natural Fibre Engineering and Technology, Kolkata, India; 3 ICAR- Central Institute for Women in Agriculture, Bhubaneswar, India; 4 ICAR-Central Research Institute for Jute and Allied Fibres, Barrackpore, India

## Abstract

Lack of appropriate donors, non-utilization of high throughput phenotyping and genotyping platforms with high genotype × environment interaction restrained identification of robust QTLs for grain protein content (GPC) in rice. In the present investigation a  BC_3_F_4_ mapping population was developed using grain protein donor, ARC10075 and high-yielding cultivar Naveen and 190 lines were genotyped using 40 K Affimetrix custom SNP array with the objective to identify stable QTLs for protein content. Three of the identified QTLs, one for GPC (*qGPC1*.*1*) and the other two for single grain protein content (*qSGPC*2.*1*, *qSGPC7*.*1*) were stable over the environments explaining  13%, 14% and 7.8% of the phenotypic variances, respectively. Stability and repeatability of these additive QTLs were supported by the synergistic additive effects of multi-environmental-QTLs. One epistatic-QTL, independent of  the  main effect QTL was detected over the environment for SGPC. A few functional genes governing seed storage protein were hypothesised inside these identified QTLs. The *qGPC1*.*1* was validated by NIR Spectroscopy-based high throughput phenotyping in BC_3_F_5_ population. Higher glutelin content was estimated in high-protein lines with the introgression of *qGPC1*.*1* in telomeric region of short arm of chromosome 1. This was supported by the postulation of probable candidate gene inside this QTL region encoding glutelin family proteins.

## Introduction

Malnutrition is responsible for about 24,000 deaths per day worldwide^1^. Rice is staple food for more than half of the world population. It has a significant contribution in daily calorie-intake as millions of poor families depend mainly of rice for their nutrition. Rice supplies abundant carbohydrate as its kernel constitutes mainly of starch (>80%) but protein (7–8%) is the source of concern. However, the protein quality measured by protein digestibility index and amino acid composition is the best among cereals^2^, which makes it preferable for the food and feed industries. Efforts were made during past three decades by rice breeders to improve the protein content in rice grain, but significant and stable improvement could not be achieved due to the involvement of many small effect genes/quantitative trait loci (QTLs) substantially affected by environment. The QTLs for grain protein content (GPC) in rice have been identified in almost all chromosomes, though majority of them are present on chromosomes 1, 2, 6, 7, 10 and 11^3–13^. But multi-environmental stable and robust QTL for this trait was rare. This was due to the lack of high throughput genotyping platform leading to low density linkage map, low population size, lack of high throughput phenotyping procedure and lack of validation in different cropping season and environments. Moreover, this trait is not only governed by additive gene effect but also significantly influenced by the complex gene interaction including dominance, epistatic and genotype × environment interaction (G EI) component effects as realized by many researchers^12,14,15^. But, in spite of quite high probability of getting epistasis and GEI-QTLs, no notable epistatic or multi-environment trial QTL (MET-QTL) was detected in rice for this trait. With the recent advancements in rice genomics research, more robust and reproducible markers such as single nucleotide polymorphic (SNPs) markers have been utilized to make SNP chips of various magnitude, i.e. on medium density Illumina’s rice platform^16–18^, high density 50 K Illumina Infinium array platform (RiceSNP50)^19^ and Affymetrix custom array such as 44 K and 50 K SNP chips platform in rice^20,21^. In addition, Near Infrared (NIR) spectroscopy has been used by researchers to screen large number of germplasm for protein content in several cereals^22–24^ and in high throughput phenotyping of breeding lines^25^.

In bi-parental mapping, population for detection of robust QTL for a particular trait required significant differences of two parents for that trait. For detecting QTLs for GPC, rarely very high protein genotype and low protein counterpart had been used which restricted trait variability and availability of robust QTL. Several rice germplasm with high GPC have been identified over the environments^26^. They however were low yielder and had many undesirable features. Backcross breeding could be an effective approach for minimizing the undesirable effects coming from un-adapted donor parents^4,27^. Backcross population is not only useful for detecting robust QTLs but also to generate introgression lines for use as pre-breeding lines or as high yielding elite cultivars. The advanced backcross QTL (AB-QTL) analysis has been successfully employed in detecting and transferring QTLs from un-adapted germplasm into advanced breeding lines in many plant species^28–32^. In rice, AB-QTL analysis has helped to detect many QTLs for several grain quality traits^33^. But the use of two diverse parents (with regard to origin, nature, type and adaptability) often poses many problems such as lack of proper chromosomal pairing, pollen sterility in backcross lines leading to segregation distortion (SD) etc., Zhan and Xu^34^ suggested that being the potential evolutionary force, the SD loci should be effectively utilized in mapping genes using appropriate packages. Among the statistical packages utilized for mapping QTLs, a SAS-based programme Proc QTL, QTL IciMapping V4 and DistortedMap handle SD markers safely and effectively to identify regions influencing trait expression^35–38^. Inside the putative or multi-environment QTLs region, functional genes which ultimately governed the phenotype were found using bioinformatics tool in previous studies on rice^39^. In the present study high genetic variability governed by high protein donor followed by high throughput SNP-array based genotyping were exercised with the aim of detection of robust QTLs for grain protein content with plausible influence of epistasis and genotype × environment interaction. This investigation also explored the scope of high throughput phenotyping using NIR spectroscopy to validate stable QTLs in advanced near isogenic line (NIL) population. Finally it focused on the delineation of QTLs loci to find functional genes inside QTLs and tried to associate them with higher protein and protein fraction content in the selected stable high protein introgressed (NILs) over the environments.

## Results and Discussion

### Phenotypic analysis

**Figure 1:**
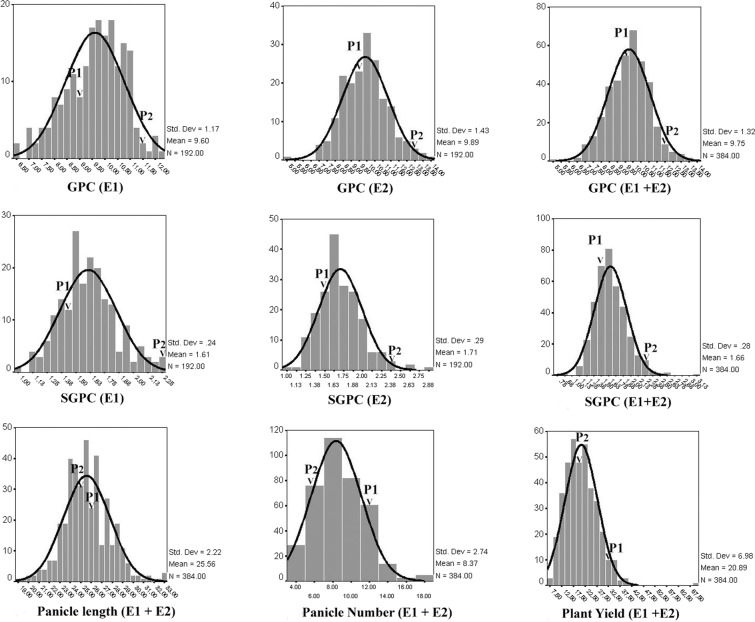
Distribution of backcross derived mapping population (BC_3_F_4_) from ARC10075/Naveen for grain protein content (GPC) and single grain protein content (SGPC) in individual environments (Env.1 and Env.2) and distribution for GPC, SGPC, panicle length, panicle number/plant and plant yield over the environments (*Env*.1 + *Env*.2) (Note: P1: Naveen, P2: ARC10075, E1: *Env*.1 (*Kharif season* 2013), E2: *Env*.2 (*Rabi season* 2014)).

ANOVA for plant height (cm) (PH), maturity duration (MD), number of panicles/plant (PN), panicle length (cm) (PL), grains/panicle (GRAIN), 100 grain weight (g) (GWT), plant yield (g) (PY), grain protein content (%) (GPC), single grain protein content (mg/g) (SGPC) in both *kharif* 2013 (*Env*.1) and *rabi season* 2014 (*Env*.2) individually and over the seasons (*Env*.1 + *Env*.2) revealed the significant variation in population for all the traits (Supplementary Table 1). High heritability (*h*^2^ = 0.75–0.78) of GPC in individual environment was observed. But this was moderate to low (*h*^2^ = 0.45) across environments calculated from pooled data. In contrary, SGPC revealed relatively higher heritability (*h*^2^ = 0.55) over the environments (Supplementary Table 1). Moreover, higher phenotypic variance of SGPC also indicated its suitability for QTL analysis. These facts indicated that SGPC was environmentally more stable than the percent protein content and therefore, transfer of this trait could be more feasible. Except PY and GWT all other traits followed normal distribution and both absolute values of skewness and kurtosis were less than 1.0, indicating suitability of data for QTL analysis (Supplementary Table 2). Transgressive segregation was observed for all traits, suggesting possible existence of multiple QTLs and QTL × QTL interaction or epistatic interaction. Transgressive segregation was observed in both directions of normal distribution for GPC and SGPC (Fig. 1). This indicated that both the parents may contribute to the QTL analysis of these traits. GPC and SGPC were not significantly (*p* < 0.01) correlated with PY in two seasons and over the seasons (Supplementary Table 3). But both these traits were significantly (*p* < 0.01) negatively associated with GRAIN which was positively associated with PY in both the seasons and over the environments. Path coefficient analysis (Supplementary Table 4) also revealed most significant direct effect of PN and GRAIN on PY, while no significant effect of GPC and SGPC was observed on PY.

**Figure 2:**
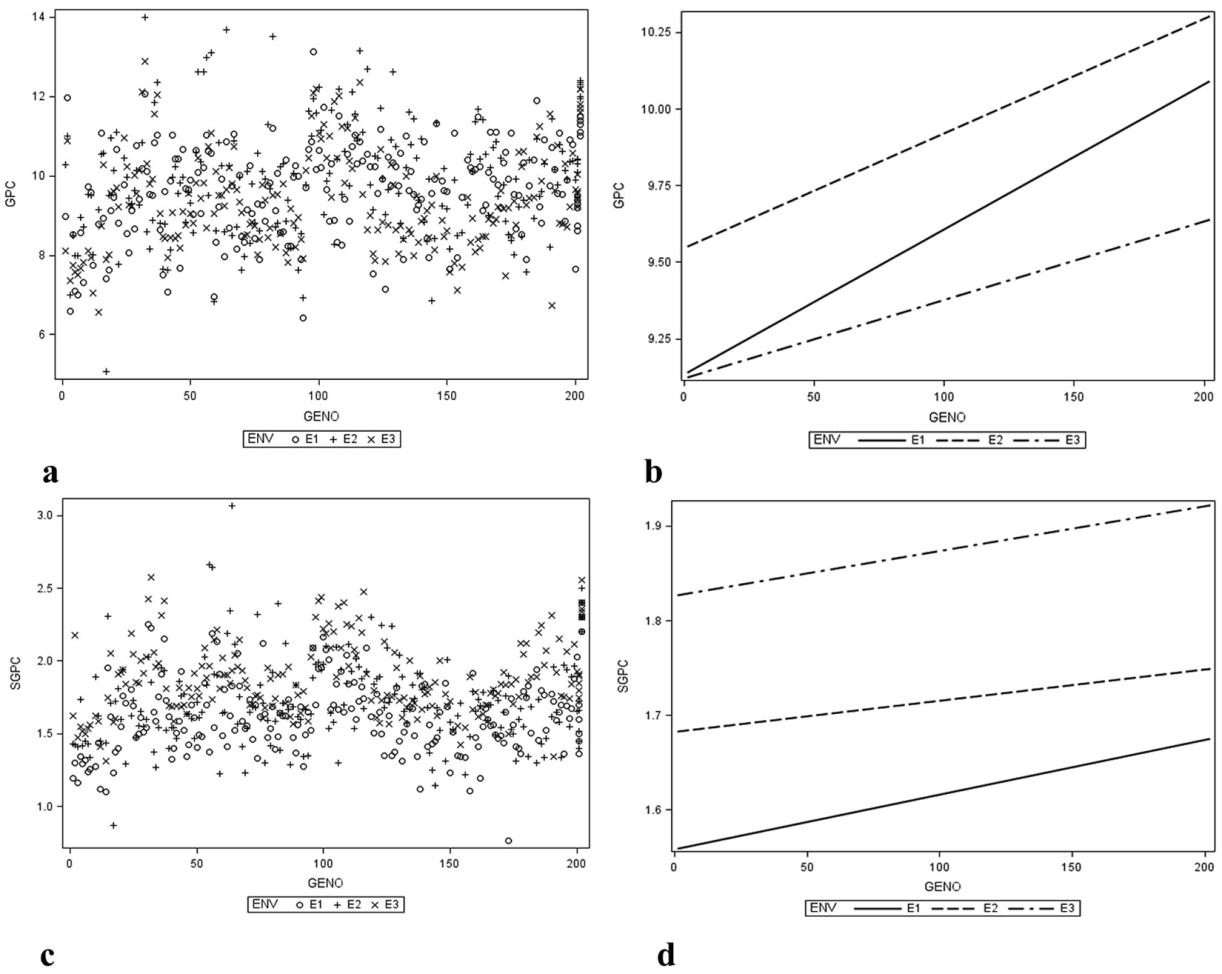
Scatterplot and trendline of mapping population for GPC and SGPC in three environments (*Env*.: E1: *kharif* 2013, E2: *rabi* 2014, E3: *kharif* 2014), viz (**a**) Scatterplot of genotype vs. GPC, (**b**) Trendline genotype vs. GPC, (**c**) Scatterplot of genotype vs SGPC, (**d**). Trendline genotype vs. SGPC.

Analysis of variance revealed significant differences (*p* < 0.001) of genotypes, environment and genotype × environment interaction for grain protein content (GPC) with nearly similar trend for single grain protein content (SGPC) in genotype (G) and environment (E) (*p* < 0.001) as well as G x E (*p* < 0.01). The significantly higher (*p* < 0.001) mean GPC of mapping population was observed in *rabi season* 2014 (*Env*.2) as compared to both the *kharif seasons* (*Env*.1 and *Env*.3). Comparative lower (*p* < 0.001) average SGPC was also found in *Env*.1 than in the *Env*. 2. Better water and nutrient management and higher light intensity in *rabi season* might have contributed to better grain filling and protein content in rice. ARC10075 had higher GPC and SGPC values than the control. Hence, ARC10075 and environment *Env*.2 were considered as reference combinations for identifying the best genotype in any specific environment. Lines, PLN-32, PLN-64, PLN-58 and PLN-56 in *Env*.2 were found superior in GPC while PLN-64 was also found superior in SGPC in *Env*. 2. Interaction plots and ANOVA suggested that the genotype × environment interaction effects were significant (*p* < 0.01) for both GPC and SGPC. The trend lines (Fig. 2) also showed that for both GPC and SGPC, all three environments were not parallel. Therefore, the presence of genotype × environment interaction effect was obvious.

### Distribution of SNPs in chip, genotyping and linkage analysis

**Figure 3:**
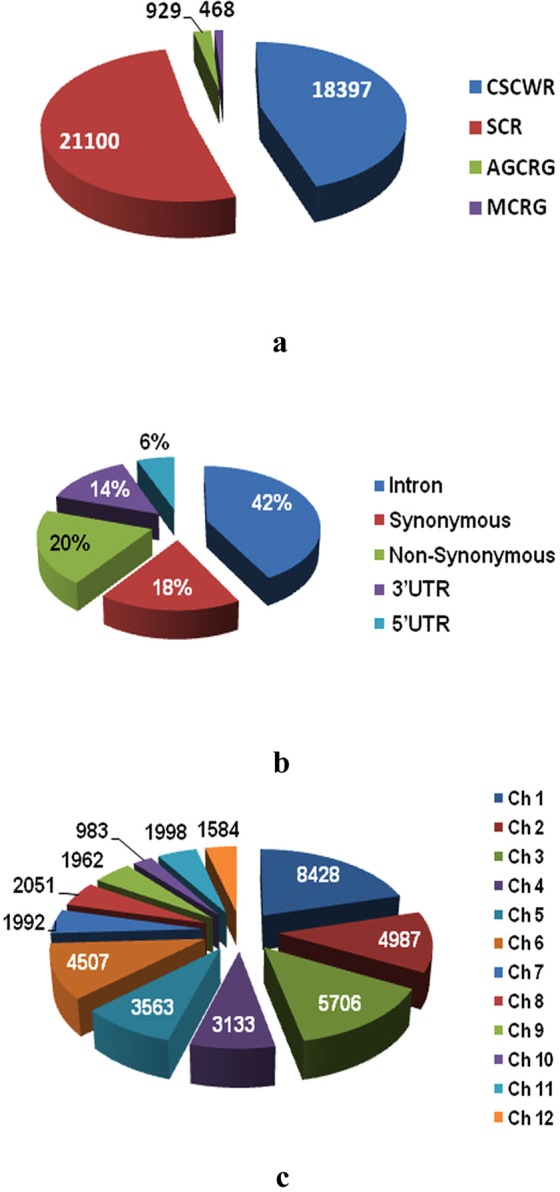
(**a**) Distribution of SNPs on different type of genes in SNP Chip, (**b**) Different types of SNPs in chip, and (**c**). Distribution of SNPs in 12 rice chromosomes (Note: SCR: single copy genes were unique to rice, CSCWR: conserved single-copy genes were common to wheat and rice, MCR: multi-copy rice gene, AGCR: agronomically important cloned rice genes; Ch-1–12: chromosome 1 to chromosome 12).

Among the four types of genes used for the 40,894 SNP chip designing, majority (96.6%) were single copy (SC) genes. The rest were from agronomically important cloned rice genes (AGCR) (2.27%) and multi-copy rice (MCR) (1.14%) genes. Further, 21100 (51.6%) single copy genes were unique to rice (SCR) and 18397 (45%) conserved single-copy genes were common to wheat and rice (CSCWR) (Fig. 3a). This SNP chip had 38% SNPs from exons, 42% from introns and 20% from 5′ and 3′UTR regions (Fig. 3b). The SNPs from exon regions could be further classified into non-synonymous (20% of total SNPs) and synonymous (18% of total SNPs) types. The non-synonymous SNPs are important for detection of probable functional genes for the trait concerned. The presence of large number of these SNPs, makes this chip more effective for associating genotypes with the desired phenotype, i.e. high protein content. Overall, the SNPs were distributed among all 12 rice chromosomes with an average of one SNP per 9.54 kb (Fig. 3c). The number of SNPs varied from 983 (chromosome 10) to 8428 (chromosome 1) with an average of 3407.83 per chromosome.

All the genotypes passed the development quality check (DQC) with a high cut-off value of > 0.82, and the maximum DQC value was 0.99. Except for one sample, all others possessed high genotyping call rates of > 95%, with an average of 99.9%. Out of the 40894 markers in SNP chips, 5492 SNPs accounting 13.43% were found to be homo-polymorphic between ARC10075 and Naveen. These markers were highly informative as many of them were located inside genes. The remaining 82.96% (33925) and 1.66% (680) were non-polymorphic and hetero-polymorphic, respectively. Only 506 SNPs accounting 1.24% were not detected in this assay (GAP) (Supplementary Table 5). GTC Software efficiently separated homozygous and heterozygous cluster (Supplementary Fig. 1). The proportion of genome of Naveen in backcross derived lines varied from 46.88 to 95.62% with an average of 81.8% based on homo-polymorphic SNP markers. The proportion of genome of the donor ARC10075 varied from 2.97 to 40.37% (average 13.06%). The rest genome with an average of 5.12% was heterozygote among backcross derived lines. Out of the homo-polymorphic markers between the parents, 87% showed segregation distortion (SD) through χ^2^ test (*p* > 0.01) and were distributed on all the 12 chromosomes. Segregation distorted markers occurred due to unwanted selection pressure imparted by pollen sterility, incompatability, epistatic and environmental interaction, etc. We employed one accession (ARC10075) as donor for high GPC which was collected from North-eastern part of India, which is considered as the secondary centre of origin for rice. Assam rice collection (ARC) represents diversity of this region. Some of the germplasm belonging to the part of the country adjoining Mayanmar, China, and Indonesia have many traits intermediate to those of *indica* and *japonica*. Therefore, chances of having sterility were high as observed frequently in inter sub-specific crosses, especially in backcross progenies which led to segregation distortion (SD). In general, distorted markers did not have much effect on the position and effect estimations of QTL; moreover, their effects can be ignored in large-size mapping populations^40,41^. In the previous studies^4,41^ large number of markers (40–55%) showing SD were successfully utilized to map grain quality traits. In traditional linkage mapping, there is all likelihood of losing all these informative markers in QTL analysis. In the present study, we handled SD markers along with non-SD markers using ‘SDL mapping’ in the QTL IciMapping V4 software which helped in restoration of order and position of the distorted markers to safely use in QTL detection. By employing these options of mapping, we could use all available markers, whether Mendelian or otherwise and could save valuable resources. A high density linkage map with 12 linkage groups on 12 rice chromosomes was generated. The average genetic to physical distance of 1 cM = 0.2 Mb. The total map distance was 2480 cM with an average 0.46 cM marker-interval. Through DistortedMap v.1 software, it was found that all markers were qualified for SD mapping analysis. Although little higher average (0.67 cM) marker distance was noticed which could be the effect of epistatic SD locus.

### Single environment QTLs

**Figure 4:**
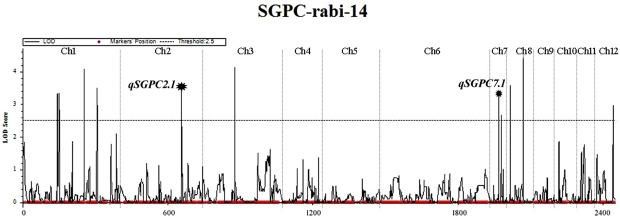
QTLs detected for SGPC in *rabi season* (*Env*.2) showing common QTLs *qSGPC2*.*1* and *qSGPC7*.*1* found in also in *kharif season* (*Env*.1).

A total of 14 main effect additive single environment QTLs for GPC and SGPC were detected by inclusive composite interval mapping (ICIM). Three of them were found in more than one environment (Table 1). Compared to GPC, more number of additive QTLs were detected for SGPC in single and multi-environment. Previously also researcher^42^ did not find any consistent environmentally stable QTLs for GPC, but detected stable QTLs for protein index (PI) which was almost identical with SGPC, used in the present study. In *kharif* season 2013 (*Env*.1), one QTL (*qGPC1*.*1*) at 11 cM position was identified for GPC on chromosome 1 with a logarithm of odds ratio (LOD) value of 3.83 which explained 13.86% phenotypic variance. In this environment, four other QTLs for SGPC were identified. One of the pleotropic QTL (*qSGPC1*.*1*) shared the same position with *qGPC1*.*1* explaining 10.37% phenotypic variance with a LOD value of 2.9. The other three QTLs for SGPC (*qSGPC2*.*1*, *qSGPC7*.*1*, *qSGPC11*.*1*) had LOD values of 3.32, 3.31 and 2.88 with 6.7%, 7.68% and 6.42% phenotypic variance explained (PVE), respectively. In *rabi* season 2014 (*Env*.2), still higher number of QTLs for both GPC and SGPC were detected. Of the two QTLs for GPC, one (*qGPC1*.*1*) was common with the previous environment (*Env*.1) explaining 13.85% phenotypic variance with LOD value of 4.02. The new putative QTL (*qGPC 2*.*1*) was detected at 170 cM position on chromosome 2 which had 17.35% PVE with LOD value of 3.19. Eleven QTLs for SGPC were found in *Env*.2 on chromosomes 1, 2, 3, 7, 8, and 12. Two of them were common with previous environment (*Env*.1). They were *qSGPC2*.*1* and *qSGPC7*.*1* with LOD values of 3.53 and 3.33, respectively which explained 14.64% and 7.81% phenotypic variance (Fig. 4).

**Table 1:** Main effect additive QTLs for GPC and SGPC in rice in two environments (*Env*.1 and *Env*.*2*).

Trait/QTL	Environment	Chromosome	Left marker	Right marker	Start (Mb)	End (Mb)	Peak marker	LOD	PVE (%)	Add	Position (Mb)	Type of SNP	Gene function
*qGPC1*.*1*	*Env*.*1*	1	Affx-93237905	Affx-93229368	0.61104	1.11104	CSCWR_Os01g02590__61041	3.832	13.855	−0.426	0.86104	non-synonymous SNP, resides in gene	Receptor-like kinase, putative, expressed
*qSGPC1*.*1*	*Env*.*1*	1	Affx-93237905	Affx-93229368	0.61104	1.11104	CSCWR_Os01g02590__61041	2.897	10.37	−0.083	0.86104	non-synonymous SNP, resides in gene	Receptor-like kinase, putative, expressed
*qSGPC2*.*1*	*Env*.*1*	2	Affx-93260438	Affx-93236905	5.16506	6.16506	CSCWR_Os02g10740_65058	3.316	6.703	0.059	5.66506	resides in gene, synonymous SNP	Calcium-binding mitochondrial carrier CBG00135, putative, expressed
*qSGPC7*.*1*	*Env*.*1*	7	Affx-93225742	Affx-93256949	22.1975	22.2975	SCR100_Os07g37440_17971	3.51	7.678	0.067	22.2475	resides in gene, resides in intron	Hypothetical protein
*qSGPC11*.*1*	*Env*.*1*	11	Affx-93232878	Affx-93212320	3.73772	3.83772	SCR200_Os11g07480_87716	2.873	6.424	0.076	3.78772	non-synonymous SNP, resides in gene	WD domain, G-beta repeat domain containing protein, expressed
*qGPC1*.*1*	*Env*.*2*	1	Affx-93237905	Affx-93229368	0.81104	0.91104	CSCWR_Os01g02590__61041	4.017	13.851	−0.581	0.86104	non-synonymous SNP, resides in gene	Receptor-like kinase, putative, expressed
*qGPC2*.*1*	*Env*.*2*	2	Affx-93221488	Affx-93245529	9.47632	10.4763	SCR200_Os02g17350_76316	3.186	17.353	0.923	9.97632	resides in gene, synonymous SNP	VHS and GAT domain containing protein, expressed
*qSGPC1*.*2*	*Env*.*2*	1	Affx-93230672	Affx-93212941	39.0164	39.1164	SCR100_Os01g66690_66361	3.309	18.463	0.492	39.0664	resides in gene, synonymous SNP	Gene encoding ZIP4/SPO22
*qSGPC1*.*3*	*Env*.*2*	1	Affx-93228332	Affx-93233227	8.30788	8.40788	CSCWR_Os01g14920__57875	4.07	16.401	0.481	8.35788	resides in 5′ UTR, resides in gene	Zinc knuckle family protein, putative, expressed
*qSGPC2*.*1*	*Env*.*2*	2	Affx-93256429	Affx-93260438	5.61506	5.71506	CSCWR_Os02g10740_65058	3.528	14.636	0.54	5.66506	resides in gene, synonymous SNP	Calcium-binding mitochondrial carrier CBG00135, putative, expressed
*qSGPC3*.*1*	*Env*.*2*	3	Affx-93253793	Affx-93260929	35.3227	37.3227	CSCWR_Os03g64360_22659	4.115	14.653	0.542	36.3227	resides in gene, resides in intron	Putative expressed gene
*qSGPC7*.*1*	*Env*.*2*	7	Affx-93225742	Affx-93256949	22.1975	22.2975	SCR100_Os07g37440_17971	3.328	7.813	0.091	22.2475	resides in gene, resides in intron	Hypothetical protein
*qSGPC8*.*1*	*Env*.*2*	8	Affx-93259293	Affx-93258892	0.90055	1.00055	SCR200_Os08g02400_50552	4.548	23.547	0.336	0.95055	resides in gene, resides in intron	40 S ribosomal protein S13, putative, expressed
*qSGPC12*.*1*	*Env*.*2*	12	Affx-93257146	Affx-93240174	2.27059	2.37059	CSCWR_Os12g05230_20586	2.966	14.486	0.531	2.32059	resides in 3′ UTR, resides in gene	ATP-dependent RNA helicase, putative, expressed

Apart from GPC and SGPC, QTLs were detected for other traits such as panicle length (PL), panicle number/plant (PN), grains/panicle (GRAIN) which were normally distributed. In *Env*.1, 15 putative QTLs (PL-1, PN-13, GRAIN-1) distributed among chromosomes, 1, 2, 4, 5, 8, 9, 10, 11 and 12 explaining 6% to 32.5% phenotypic variance (PVE) (Supplementary Table 6) and in *Env*.2, 5 putative QTLs (PL-3, GRAIN-2) distributed in chromosomes, 1, 2, 6 and 7 with 6.1% to 24.67% PVE (Supplementary Table 7) were detected. But none of them was detected over the environments. Simple interval mapping which is based on maximum likelihood may not be as efficient as ICIM, but it can provide information on small effect QTLs independent from variance of other QTLs. IM was used to identify QTLs for GPC and SGPC in the present investigation. Multi-environmental consistent QTLs such as *qGPC1*.*1* and *qSGPC2*.*1* which were identified by ICIM, were also found in interval mapping. Position of another consistent QTL, *qSGPC7*.*1* was little shifted in this analysis. Apart from them single environment putative QTLs, *qSGPC1*.*1*, *qSGPC11*.*1*, *qSGPC1*.*2*, *qSGPC1*.*3*, *qSGPC3*.*1*, *qSGPC7*.*2*, *qSGPC8*.*1*, *qSGPC8*.*2*, *qSGPC12*.*1* which were detected by ICIM, were also found through IM. In addition 14 other putative QTLs on chromosome 1, 3, 4, 8 and 11 were found by this analysis (Supplementary Table 8).

### Epistatic QTLs and MET- QTLs for GPC and SGPC

**Figure 5:**
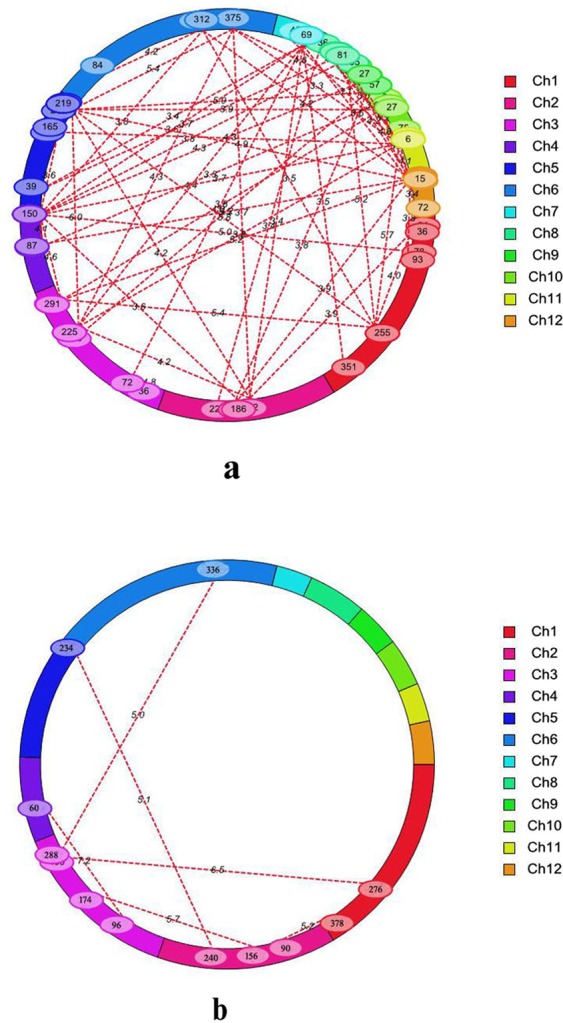
(**a**) Cyclic diagram of epistatic QTLs (ep-QTL) for single grain protein content (SGPC) in rabi season 2014 (*Env*.2) and (**b**) epistatic × environmental (aae) interaction QTLs for grain protein content (GPC). The dotted lines indicate the interacting SNP marker pairs situated on the same or different chromosomes with corresponding LOD scores owing to their epistatic effects. Marker position (cM) is mentioned inside the oval located on chromosome. One common ep-QTL in *kharif season* 2013 (*Env*.1) is located between 6 cM position on chromosome 11 and 255 cM position on chromosome 1 (**a**).

The epistatic interaction can not be ignored because such attempt may lead to underestimation of total genetic effects of a trait. The proper detection of the direction of epistatic interaction, i.e. synergistic or non-synergistic effect on other QTLs can guide the breeder to introgress multi-QTLs for one or many traits. Epistatic interaction for grain protein content was reported in other cereals^43,44^. Except rare instances^45^ no significant epistatic QTL was detected so far in rice for this trait. In the present study although no digenic epistatic interaction QTLs (ep-QTLs) were identified for GPC, 11 in *Env*.*1* (Supplementary Table 9) and 62 in *Env*.*2* (Supplementary Table 10) such QTLs (ep-QTLs) were detected in SGPC. Except one ep-QTL pair no other was repeatable over the environments. This epistatic QTLs pair on chromosome 11 in 6 cM region showed epistatic interaction with one pair of ep-QTL on chromosome 1(Fig. 5a). The peak SNP markers, SCR100_Os01g40720_34486 and SCR100_Os11g08270_33249 for this ep-QTL were non-synonymous, resided inside genes. Similar sign indicated that this epistatic effect contributed positively towards the additive value and could increase the phenotypic value independently from main effect QTLs of SGPC.

**Figure 6:**
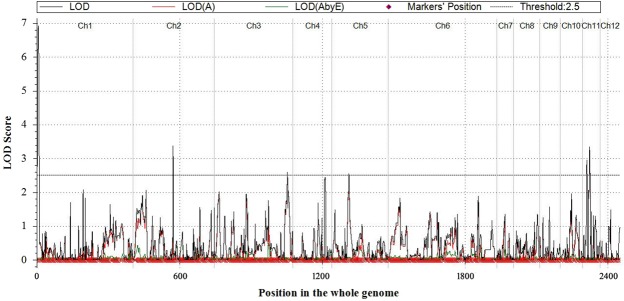
Multi-environment trial QTLs (MET-QTLs) detected for grain protein content (GPC) in rice in threshold LOD score 2.5.

The GPC was found to be highly influenced by environment. Therefore, selection of breeding lines based on only the phenotypic effect (which is significantly contributed by environment) can be misleading. Therefore, the objective of our experiment was to identify main effect additive QTLs for GPC and SGPC in more than one environment. In the present investigation we detected three such QTLs, one for GPC (*qGPC1*.*1*) and two for SGPC (*qSGPC2*.*1* and *qSGPC7*.*1*). Earlier *qPC-1* was detected in all the three studied environments, *qPC-10* in two environments, and the rest 8 QTLs in only one environment^12^. Therefore, in spite of high phenotypic variation, stably inherited QTLs such as *qPC-1* and *qGPC1*.*1* are present in rice. The stable genomic region inside these QTLs can guide the selection for these traits more efficiently. In addition, genotype × environmental interaction QTLs are also important as they significantly influence the total phenotypic variance and additive effect of the main effect QTL located inside or near to them. Although there are some reports in other cereals like wheat^43,46^, no multi-environment trial QTL (MET-QTL) was reported earlier for rice GPC. We found significant G × E interaction effects both for GPC and SGPC. Five MET-QTLs for GPC (Fig. 6) and six MET-QTLs for SGPC were detected (Table 2). Among them one MET-QTL for GPC was located inside the main effect additive QTLs (*qGPC1*.*1*) and another was adjacent to the main-effect putative QTL (*qGPC2*.*1*). Two MET-QTLs (*Eq-GPC1*.*1* and *Eq-SGPC1*.*1*) were pleotropic for these two traits. They were located inside the main effect QTLs (*qGPC1*.*1*, *qSGPC1*.*1)*. Among other MET-QTLs, three were located on the main effect QTLs. They were *Eq-SGPC2*.*1*, *Eq-SGPC7*.*1* and *Eq-SGPC11*.*1*. The additive values of these MET-QTLs were showed similar sign with the main effect QTLs. Therefore, they had positive effect on the total additive value of this trait. Hence, although these loci have GEI effect, they can be safely used in the molecular breeding programme. On the other hand, it also indicated that all these MET-QTLs had significant positive effect on positive allele which improved the phenotypic expression leading to higher GPC and SGPC in rice in favourable environments. Finally the epistasis × environment interaction effect (aae) was an important component of QTL × environment (QE) interaction effects. In MET analysis 6 pair (Supplementary Table 9) and 48 pair (Supplementary Table 12) of QTLs associated with GPC (Fig. 5b) and SGPC, respectively, were found with epistatic effects (aa) and epistasis × environment (aae) effects. But none of them was found associated with the main effect QTLs. One main epistatic QTL was adjacent to the main effect QTL (*qSGPC11*.*1*) and MET QTL, *Eq-SGPC11*.*1* on chromosome 11. In the similar position, the gene *OsAsp1* coding for aspartic acid was located. The synergistic effect of epistatic QTL on the MET-QTL and the probable functional gene *OsASP1* may significantly contribute positively to protein content in rice grain.

**Table 2:** Additive × environment interaction QTLs (MET-QTLs) for grain protein content (GPC) and single grain protein content (SGPC).

MET-QTL	Chromo-some	Position (Mb)	Left Marker	Right Marker	Peak locus	SNP information	LOD	PVE	PVE (A)	PVE (AbyE)	Add	AbyE_01	AbyE_02	Heritability (*h2*)
*Eq-SGPC1*.*1*	1	0.861041	Affx-93237905	Affx-93229368	CSCWR_Os01g02590__61041	Non-synonymous SNP, resides in gene	4.604	6.553	6.475	0.077	−0.078	0.009	−0.009	0.988
*Eq-SGPC1*.*2*	1	1.504321	Affx-93256957	Affx-93252910	SCR200_Os01g03650_04321	Resides in gene, synonymous SNP	4.289	5.394	4.310	1.084	0.057	−0.028	0.028	0.799
*Eq-SGPC2*.*1*	2	5.665058	Affx-93260438	Affx-93236905	CSCWR_Os02g10740_65058	Resides in gene, synonymous SNP	4.416	3.811	3.762	0.049	0.052	0.006	−0.006	0.987
*Eq-SGPC2*.*2*	2	26.38269	Affx-93234385	Affx-93236297	CSCWR_Os02g43720_82691	Resides in gene, resides in intron	2.797	2.622	2.599	0.023	−0.097	0.009	−0.009	0.991
*Eq-SGPC7*.*1*	7	22.24752	Affx-93225742	Affx-93256949	SCR100_Os07g37440_17971	Resides in gene, resides in intron	6.836	7.510	7.320	0.190	0.078	−0.013	0.013	0.974
*Eq-SGPC11*.*1*	11	3.787716	Affx-93232878	Affx-93212320	SCR200_Os11g07480_87716	Non-synonymous SNP, resides in gene	4.298	4.159	4.159	0.000	0.073	0.000	0.000	1.000
*Eq-GPC1*.*1*	1	0.861041	Affx-93237905	Affx-93229368	CSCWR_Os01g02590__61041	non-synonymous SNP, resides in gene	6.923	8.988	8.885	0.103	−0.427	0.046	−0.046	0.988
*Eq-GPC2*.*1*	2	9.976316	Affx-93243043	Affx-93221488	SCR200_Os02g17350_76316	Resides in gene, synonymous SNP	3.371	4.055	3.551	0.505	0.261	−0.098	0.098	0.875
*Eq-GPC3*.*1*	3	35.27895	Affx-93211998	Affx-93250371	SCR200_Os03g62388_78948	Resides in gene, synonymous SNP	2.592	2.935	2.696	0.239	0.221	−0.066	0.066	0.918
*Eq-GPC5*.*1*	5	1.237304	Affx-93260300	Affx-93258158	SCR200_Os05g03150_37304	Resides in 3′ UTR, resides in gene	2.543	2.649	2.649	0.000	0.221	−0.001	0.001	1.000
*Eq-GPC11*.*1*	11	3.787716	Affx-93232878	Affx-93212320	SCR200_Os11g07480_87716	Non-synonymous SNP, resides in gene	2.955	3.361	3.222	0.139	0.328	−0.068	0.068	0.958

### Validation of main effect QTL for GPC using high throughput phenotyping

The need of destructive sampling and tedious analysis procedure is a major bottleneck for mapping QTLs and selecting superior genotypes in segregating generations for grain protein. We attempted QTL mapping for GPC using near infrared (NIR) spectroscopy-based high throughput non-destructive protein estimation method in *kharif* 2014 (*Env*.3). Calibrated NIR spectroscopy for GPC^24^ was used to determine the apparent grain protein content in NILs (BC_3_F_5_) derived from Naveen/ARC10075. NIL population showed the normal distribution with positive skewness for apparent GPC (Supplementary Fig. 2). The GPC varied from 6.56% to 12.89% with mean value of 9.33%. We detected the same QTL (*qGPC1*.*1*) as was found through phenotyping by conventional *micro-Kjeldahl* method. It also explained almost similar phenotypic variance (12.18%) as observed in *Env*.1 and *Env*.2 (13.85%). This observation validated QTL for GPC and also established the NIR spectroscopy as the valid high throughput phenotyping tool for detection of stable QTL for GPC in rice.

### Delineation of QTL loci and identification of probable candidate genes

Normal distribution for GPC and SGPC indicated the involvement of many QTLs for grain protein in rice. However, some regulatory genes were reported to be involved in controlling GPC in seeds in many cereal crops such as barley, wheat and rice^47–50^. GWAS and candidate gene based association study identified a gene *HVNAM* controlling GPC in barley^51^. In wheat also, a high grain protein gene, *Gpc-B1* was introgressed to improve protein content without affecting grain yield^47,52^. In the present experiment, the functional genes presents inside and adjacent to the QTLs were identified (Table 3). One main effect additive QTL *qGPC1*.*1* was found stable over the environments. The peak SNP marker inside this QTL (locus- 1:611041-1111041) in *Env*.1 was CSCWR_Os01g02590__61041 which is located inside a conserved single-copy gene common to wheat and rice. This QTL interval region corresponded to a span of 186 *O*. *sativa Japonica* genes starting from *Os01G0111600* to *Os01g0119500*. This QTL interval was narrow (locus- 1:811041-911041) in *Env*.2 corresponding only 34 coding and non-coding genes staring from *Os01g0115100* to *Os01g0116000* with same peak locus as was detected in *Env*.1. Multi environmental (MET) QTLs, *Eq-GPC1*.*1* and *Eq-SGPC1*.*1* were pleotropic (locus- 1:811041-911041) inside the main effect robust QTL, *qGPC1*.*1*. Among genes located inside these QTLs one gene *Os01g0111900* (locus- 1:625986–627009) encoded glutelin family protein. This gene was located just 0.93 cM apart from the QTL peak. Inside one putative single environment QTL, *qSGPC1*.*3* (locus- 1:8307875–8407875) a gene *Sar1c* (*Os01g0254000*) encoding seed storage protein (pro-glutelin content in seed, floury endosperm) was located just 0.23 cM apart from the QTL peak (Q-TARO annotation). Adjacent to one putative QTL, *qSGPC1*.*2* (locus- 1:39016361–39116361), around 0.83 Mb upstream region, *OsAAP6* gene was present which was amino acid transporter enhancing GPC. On chromosome 2, one putative QTL, *qGPC2*.*1* (locus- 2:9476316–10476316) and in the MET-QTL *Eq-GPC2*.*1* with relatively narrow interval (locus- 2:9926316–10026316) contained two genes *Os02g0268100* and *Os02g0268300* which were 1.5 cM apart from the QTL peak. They also encoded glutelin protein. A gene cluster encoding glutelin fragment proteins and prolamin box binding factors is also found near to it. On chromosome 3, inside one putative QTL *qSGPC3*.*1* (locus- 3:35322659–37322659) and MET-QTL, *Eq-GPC3* (locus- 3:35228948–35328948) one gene *Os03g0826500* encoded anthranilate synthase alpha 1 related to higher grain protein content (Q-TARO annotation). This was located 3.01 cM and 1.1 cM apart from the peak of main QTL and MET-QTL, respectively. On chromosome 11, one putative QTL, *qSGPC11*.*1* and two MET-QTLs, *Eq-qSGPC11*.*1* and *Eq-GPC11*.*1* (locus- 11:3737716–3837716) had the peak marker SCR200_Os11g07480_87716. One gene *OsAsp1* influencing seed protein synthesis was located 0.62 Mb downstream of this QTL peak.

**Table 3:** Predicted functional genes present inside and adjacent to the main, epistatic and MET- QTLs and their distances from the QTLs peak.

*Trait/QTL*	*Environment*	Chromo-some	Peak marker	Position (Mb)	Number of genes in QTL interval	Starting - ending gene at QTL interval	Nearest functional gene (RAP DB ID/Q-TARO ID)	Gene function	Gene Position (Mb)	Distance (gene-QTL) (Mb)
*qGPC1*.*1*	*Env*.*1*, *Env*.*2*, *Env*.*3*, *MET*	1	CSCWR_Os01g02590__61041	0.861041	186	*Os01G0111600*-*Os01g0119500*	*Os01g0111900*	Glutelin family protein. (Os01t0111900-01)	0.6264975	0.23454
*qSGPC1*.*1*	*Env*.*1*									
*qSGPC7*.*1*	*Env*.*1*, *Env*.*2*, *MET*	7	SCR100_Os07g37440_17971	22.24752	28	Os07g0556500 – Os07g0558500	*Os07g0570100*, *Os07g0570300*, *Os07g0570500*	Gene cluster of three peptidase proteins	23.614	1.35
*qSGPC11*.*1*	*Env*.*1*, *MET*	11	SCR200_Os11g07480_87716	3.788	35	Os11g0175300 – Os11g0177200	*Os11g0184800 (OsAsp1)*	OsAsp1	4.34	0.62
*ep-qSGPC11*.*1*	*Epistatic in Env*.*1 and Env*.*2*	11	SCR100_Os11g08270_33249	4.333	—	—	*Os11g0184800 (OsAsp1)*	OsAsp1	4.34	0.01
*qGPC2*.*1*	*Env*.*2*	2	SCR200_Os02g17350_76316	9.976316	251	*Os02g0265700 – Os02g0281200*	*Os02g0268100*	Similar to Glutelin (Fragment). (Os02t0268100-01)	9.5814845	0.394
							*Os02g0268300*	Similar to Glutelin (Fragment). (Os02t0268300-00)	9.587016	0.389
							Os02g0252400 (RPBF)	Prolamin box binding factor	8.62	1.35
*Eq-GPC2*.*1*	*MET*	2	SCR200_Os02g17350_76316		26	*Os02g0272900*- *Os02g0274100*	*Os02g0268100*	Similar to Glutelin (Fragment). (Os02t0268100-01)	9.5814845	0.394
*qSGPC1*.*2*	*Env*.*2*	1	SCR100_Os01g66690_66361	39.06636	28	*Os01g0897700- Os01g0899100*	Os01g0878700 (OsAAP6)	Amino acid transporter, transmembrane domain containing protein	39.89	0.83
*qSGPC1*.*3*	*Env*.*2*	1	CSCWR_Os01g14920__57875	8.357875	36	*Os01g0251400- Os01g0253800*	Os01g0254000 (Sar1c)	Seed storage protein.Pro-gultelin content in seed. Floury endosperm.	8.41	0.06
*qSGPC3*.*1*	*Env*.*2*	3	CSCWR_Os03g64360_22659	36.32266	383	Os03g0840200 – Os03g0862200	Os03g0826500	Anthranilate synthase alpha 1	35.57	0.75
*Eq-GPC3*.*1*	*MET*	3	SCR200_Os03g62388_78948	35.28	32	Os03g0838400 – Os03g0826500	Os03g0826500	Anthranilate synthase alpha 1	35.57	0.29

### Analogy with previous findings on QTLs for grain protein in rice

A few QTLs identified in present study were located near or inside the QTLs and genes for GPC reported earlier. The main effect additive QTL, *qSGPC1*.*3* was located near to *qPr1* at 12 Mb region on chromosome 1^9^. On the same chromosome, another QTL, *qSGPC1*.*2* was identified at 39.07 Mb position which was very near to a reported QTL *qPC1*^5^ and cloned gene (*OsAAP6*)^48^ inside this QTL at 38.13 Mb region. One epistatic QTL, *ep-qSGPC-1* which was identified over the season was also located adjacent to *qPC-1* at 24.39 Mb region with 10.5% phenotypic variance explained (PVE)^12^. On chromosome 2, one MET-QTL, *Eq-SGPC2*.*2* was located adjacent to a main effect QTL *qPC2* for grain protein content^4,46^. On the same chromosome, one QTL *qSGPC2*.*1* identified in more than one environments and in MET analysis was detected near another major QTL *qPro-2* explaining 41% PVE for grain protein at 4.3 Mb position^10^. On chromosome 3 only one main effect QTL, *qSGPC3*.*1* detected in the present study was located near to a reported QTL, *qPC-3*^12^. On chromosome 7, 1.35 Mb downstream of one stable QTL *qSGPC7*.*1* and MET-QTL *Eq-SGPC7*.*1* (locus- 7:22197522–22297522) three gene cluster *(Os07g0570100*, *Os07g0570300*, *Os07g0570500)* encoding peptidase protein was located^53^. A few other QTLs (*qCP7*, *qPr7*, *PC7*) reported earlier^3,6,9^ were also located near the present QTL *qSGPC 7*.*1*. Another putative QTL, *qSGPC8*.*1* on chromosome 8 was located just adjacent to a QTL for grain protein content *qPro-8* at 1.2 Mb position^11^. We found another putative QTL *qSGPC11*.*1* and MET-QTL on the same position which was located very near to a QTL, *qGPC-11* detected through association mapping at 4.3 Mb position with peak SNP located on gene *OsAsp1*^53^.

### High protein elite NILs: their significance in mapping and validation of robust QTL

**Figure 7:**
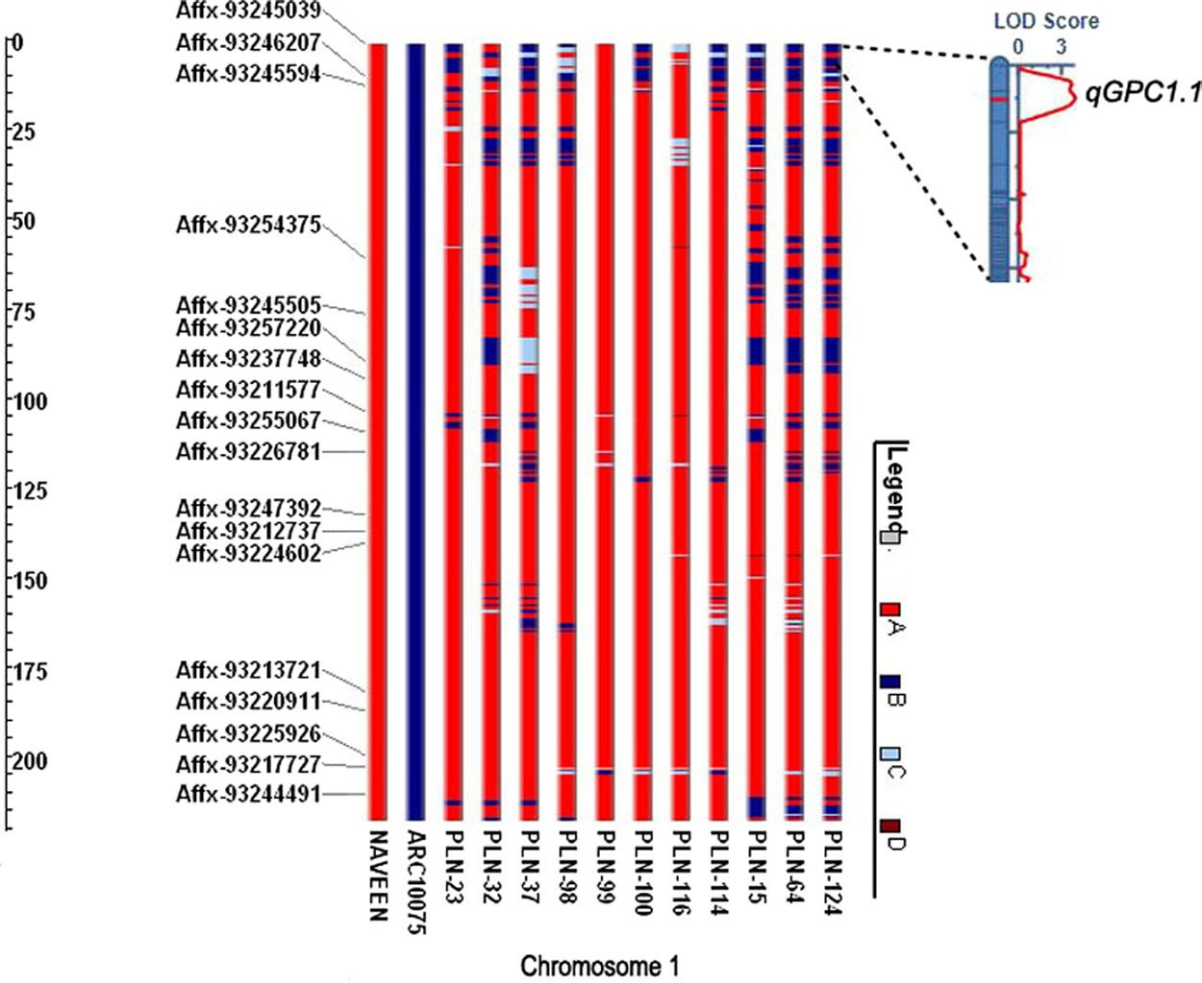
Graphical genotyping of selected high protein lines and QTL (*qGPC1*.*1*) position on telomeric region of short arm of chromosome 1 (Note: A: Naveen genome, B: ARC10075 genome, C: heterozygote, D: missing/unknown).

Seven high yielding introgression lines (BC_3_F_5_) were selected for high GPC and phenotypic resemblance with Naveen. They had comparable maturity duration (121–127 days) and plant height (108–115 cm) with Naveen (124 days, 113 cm). All lines had significantly higher GPC and SGPC in both *rabi* and *kharif* 2015 (*Env*.4 and *Env*.5). The average protein yields of these lines were also higher than those of their high yielding parent (Supplementary Table 13). These selected lines along with another three high protein lines without phenotypic resemblance with Naveen were analysed for genomic composition. They had 81–87% genome from Naveen and 10.7–16.7% from ARC10075. GGT analysis revealed that except for two all the selected high protein lines had the genomic region with the stable QTL *qGPC1*.*1* in telomeric region (~0.8 Mb) of short arm of chromosome 1 (Fig. 7). Gene (*Os01g0111900)* present in this region (locus- 1:625986–627009) synthesized glutelin protein. Significantly, except two, all high protein lines had higher (*p* > 0.01) glutelin content than the recurrent parent, Naveen. Glutelin contains essential amino acids like lysine and is the major constituent of protein body II, which is more digestible than protein body I, which contains mostly prolamins^54^. Therefore, higher accumulation of glutelin ensures better protein quality in most of these lines. It was reported earlier that improvement of grain protein content reduced the protein quality and resulted in hardening of the cooked rice grains due to increase in prolamin fraction. The ratio of prolamin to glutelin fractions ranged from 0.02 to 0.037 (Table 4). All high protein lines had similar or slightly lower values of prolamin/glutelin ratio than the high yielding variety Naveen which ensures retention of cooking quality of the introgression lines. Further, high head rice recovery (54–67%), intermediate amylose content (20–22%), alkali spreading value (3–5) and acceptable grain elongation ratio indicated good milling and cooking quality of these lines (Supplementary Table 14).

**Table 4:** Fraction of soluble protein (g per  100 g polished rice sample) in introgression lines for GPC in Naveen background and their parents.

SL no	Genotype	Albumin	Globulin	Prolamin	Glutelin	Total	Prolamin/glutelin ratio
1	ARC 10075	0.434	1.415	0.443	12.864	15.156	0.034
2	Naveen	1.406	1.02	0.244	9.297	11.967	0.026
3	PLN-23	1.48	1.575	0.333	8.889	12.276	0.037
4	PLN-32	0.988	1.283	0.24	11.058	13.57	0.022
5	PLN-37	0.44	1.263	0.352	13.519	15.575	0.026
6	PLN-98	0.823	1.507	0.365	11.61	14.306	0.031
7	PLN-99	0.798	1.28	0.296	10.584	12.959	0.028
8	PLN-100	0.565	1.292	0.356	12.49	14.703	0.029
9	PLN-116	0.283	0.9	0.242	11	12.425	0.022
	Mean	0.802	1.282	0.319	11.257	13.660	0.0283
	CD (p < 0.05)	0.06	0.11	0.08	0.35	0.41	—

### Expression profile of the functional gene located within QTL loci

**Figure 8:**
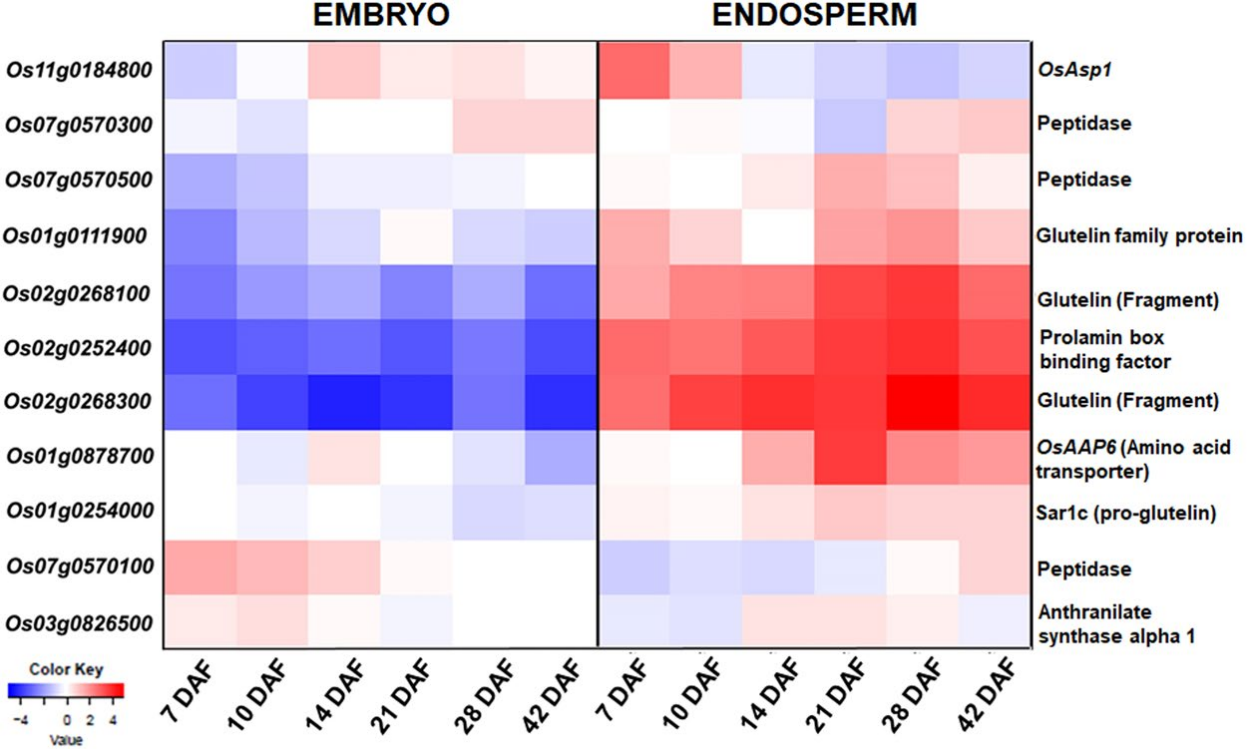
The Heat map depicting expression profiles of selected 11 genes in embryonic and endosperm-specific tissues at 7-, 10-, 14-, 21-, 28- and 42- days after flowering (DAF), respectively. (Note: The X-axis represents source and collection-time the sample used to generate the expression data while the Y-axis represents hierarchical clustering pattern. Selected 11 genes to generate the heat map are mentioned as per their RAP-ID).

Most of the probable functional genes (Table 3) inside the QTLs showed up-regulation in seed as a whole, aleurone layer, panicle tissues and root (Supplementary Fig. 3) based on RNA-seq data in Rice expression database (RED). Using RiceXPro database (RXP_0012) an expression heat map was generated to compare the gene expression profile of the 11 probable functional genes located inside QTLs in embryonic and endosperm-specific tissues at 7-, 10-, 14-, 21-, 28- and 42- days after flowering (DAF), respectively. The heat map (Fig. 8) clearly demonstrated the up-regulation of majority of the genes, including genes for enhancing storage proteins *viz*. glutelin and prolamin, preferentially at endosperm in all the time-points considered under study. Preferential up-regulation of functional genes for high GPC during endosperm development suggests higher accumulation of total protein in the selected introgression lines listed in Table 4.

## Conclusion

We found that had we discarded SD markers, the stable QTLs like *qGPC1*.*1* or *qSGPC2*.*1* and *qSGPC7*.*1* and their MET-QTLs would have remained undetected. Such QTLs detected through high throughput genotyping were not reported earlier. One of the reason could be the employment of unique germplasm in the present study which consistently showed high GPC (12%) as compared to low yielding counterpart (8%) leading to high genetic variation. The most stable QTL detected in our investigation *qGPC1*.*1* was validated in an additional environment (*Env*.3) employing high throughput phenotyping technique. Another putative QTL for SGPC (*qSGPC1*.*1*) was found pleiotropic to the former. Inside this QTL region, one gene (*Os01g0111900*) was found which encoded glutelin family protein. Physical (0.6–1 Mb) and linkage map (7–10 cM) position was highly corresponding. RiceXPro database revealed upregulation of this gene in endosperm during seed development. This was corresponded with higher glutelin content in introgressed lines. Positive correlation was reported earlier between total protein and glutelin content in rice grain^6^. In our experiment, we also found similar trend in NILs with high GPC. Fine mapping of this region using mapping population derived from high-protein-NIL × Naveen is in progress to detect tightly linked marker for marker assisted selection. Another stable QTL, *qSGPC7*.*1* was detected near to a cluster of genes encoding three peptidase proteins^53^. More than 15 genes are responsible for glutelin synthesis which accounts more than 80% of the total seed storage protein. We reported a few other probable functional genes which were located inside or adjacent to the identified QTLs for GPC and SGPC in the present study.

## Materials and Methods

### Plant materials and development of mapping population

Through evaluation of 248 germplasm we identified a few (Supplementary Table 15) which consistently showed high protein content in both brown and polished rice. But they were mostly low yielders (<3000 kg/ha grain yield). The ARC 10075 was one of them with an average 12–13% GPC in brown rice^25^. This germplam was crossed with a high yielding (4500 kg/ha grain yield) popular variety, Naveen with an average 8% GPC. F_1_ plants were backcrossed with the recurrent parent, Naveen to get 25 BC_1_F_1_ lines. Finally, 200 lines of BC_3_F_4_ and BC_3_F_5_ were developed by three consecutive backcrossing followed by single seed descent method.

### Field experiments and phenotypic evaluation

One hundred ninety lines from the backcross population (BC_3_F_4_) were planted in three rows, 15 plants in each row with 20 cm row to row and 15 cm plant to plant spacing in augmented randomized block design along with replicated checks (Naveen and ARC 10075) following standard package of agronomic practices in *kharif* season 2013 (*Env*.1), *rabi* season 2014 (*Env*.2) and *kharif* season 2014 (*Env*.3) at the experimental farm of ICAR-National Rice Research Institute, Cuttack, Odisha, India. Ten randomly selected plants were used to study agronomic traits, including plant height (PH), maturity duration (MD), number of panicles/plant (PN), panicle length (cm) (PL), number of grains/panicle (GRAIN), 100 grain weight (GWT) and plant yield (PY). Seven selected introgression lines (BC_3_F_5_) with high GPC across the three environments and phenotypic resemblance with the recurrent high yielding parent, Naveen were again raised in replicated plots (25sq m) in *kharif* (*Env*.4) and *rabi* season 2015 (*Env*.5). Nitrogen, phosphorus, and potassium were supplied @ 80 kg, 60 kg, and 40 kg per hectare, respectively in *Env*.4 and @ 120 kg, 60 kg and 60 kg per hectare, respectively in *Env*.5. Phosphorus (as single super phosphate) was applied as a basal dose, and half of the total nitrogen (as urea) and potassium (as muriate of potash) were applied in basal and rest half were applied in two equal doses at 30 days after transplanting and at 50% flowering. The grain yield from the 25 sq m plot was converted to kg/ha.

### Estimation of grain protein content

The GPC of all entries in *Env*.1 and *Env*.2 and also from the replicated large plots of selected lines in *Env*.4 and *Env*.5 were determined by the standard *micro-Kjeldahl* method^55^ by taking ten grains of brown rice (grains devoid of husk, but with the brown bran layer intact). The grain protein content was calculated by multiplying percent nitrogen content by 5.95. Single grain protein content (SGPC) was estimated on weight basis (mg/g) from the average protein content of 10 grains. Samples of known values for GPC of these lines and other germplasm were used in calibration and validation of NIR spectrophotometry for GPC in brown rice in our laboratory^24^. The apparent grain protein content of mapping population in *kharif season* 2014 (*Env*.3) was estimated using 15 g dehusked grain in calibrated NIR spectroscopy.

### Fractionation of grain protein and estimation of grain quality traits

Extraction of rice proteins was performed by standard protocol^56^. Rice flour (6–7 g) was defatted with n-hexane. Standard steps were followed to separate protein fractions in the order of albumins, globulins, prolamins and glutelins. The extracted proteins were freeze-dried and stored at −70 °C. The protein content of each fraction was measured according to Lowry *et al*.^57^. The amylose content was measured as par standard procedure^58^. Briefly,100 mg sample was wetted with 1 ml ethanol followed by addition of 9 ml 1 N NaOH with shaking and placing the tube in a boiling water bath for 10 min. After adding 1 ml 1 N acetic acid and 2 ml Iodine reagent, the volume was made to 100 ml with water and the absorbance was measured at 620 nm. Gelatinization temperature was indirectly estimated in terms of the extent of alkali spreading value (ASV) measured using a seven-point scale ranging from score-1 (least spread) to score-7 (highest spread)^59^. The analysis of cooked rice elongation, CRE % = (ACL − BCL)/BCL × 100) (ACL: after cooking length, BCL: before cooking length) and other cooking parameters were done as described by Wang *et al*.^60^.

### Statistical analysis

The phenotypic data were subjected to analysis of variance, genotypic and phenotypic coefficient of variances, genetic advance and heritability by using statistical package WINDOSTAT version 8.6, Indostat Service, Hyderabad. Heritability (*h*^*2*^) in broad sense is calculated from σ_g_/σ_p_ where σ_g_ is genotypic variance and σ_p_ is phenotypic variance. Phenotypic data were statistically analysed and the normal distribution of phenotypic data was verified by K-S test at level of *α* = 0.01 by using software, SPSS version 15.0 (SPSS, Chicago, IL, USA). For genotype × environment interaction studies, ANOVA was performed considering independent variables viz. genotype, environment, blocks within environment and genotype × environment as fixed effects and GPC and SGPC as the response variables using PROC GLM following the standard procedures^61,62^. Graphs were plotted using PROC SGPLOT procedure of SAS 9.3 software. The t-test was employed for detection of significant differences if any for mean GPC and SGPC in multi-environments.

### SNP array design and validation

Seventy one mer 50,000 SNP sequences were downloaded from OryzaSNP@MSU databases (http://rice.plantbiology.msu.edu). These SNPs were found uniformly distributed throughout the 12 chromosomes having good representation from coding and UTR regions. They were taken mostly from single copy (SC) genes and multi-copy rice (MCR) genes and the rest from agronomically important cloned rice genes (AGCR)^21^. The SNP sequences were shared with Affymetrix Bioinformatics team at Santa Clara, California, US for *in*-*silico* selection of markers for chip design. *In-silico* validation of the assay involved preliminary screening of the designed array file for each selected SNP. Both forward and reverse probes of each SNP were assigned with p-convert values, derived from a random forest model to predict the probability of SNP conversion on the array. The model considers factors including the probe sequence, binding energy and expected degree of non-specific hybridization to multiple genomic regions. SNP probes with high p-convert values are expected to convert on the SNP array with a high probability. Potential probes were designed for each SNP in both the forward and reverse direction, each of which was designated as ‘recommended’, ‘neutral’, or ‘not recommended’ based on p-convert values through which the SNP data sets were easily filtered. Thus, SNP probes were designed by screening 50,000 SNP loci of which an extremely high proportion of 40, 894 loci (90.8%) showed high-quality scores with p-convert values of >0.40, and the vast majority of them having p-convert values of >0.6, which were successfully synthesized on the array chip. The SNPs that were highly repetitive in the genome and contained ambiguities were removed. The resulting SNPs, selected for uniform spacing across the genome not having any other SNP, indel, translocation within 10 bp were selected for high resolution mapping of genetic loci in complex traits.

### Genomic DNA preparation, SNP genotyping, allele calling and data analysis

Genomic DNA was extracted from young leaf tissues of 10 seedlings of parental lines and each of the 190 lines using CTAB method^63^. The quantity and quality of genomic DNA of each sample was determined using *nano-drop* spectrophotometer and 1% agarose gel. The samples with OD_260_/OD_280_ > 1.8 and OD_260_/OD_230_ > 1.5 and more than 10 Kb intact genomic DNA were used for SNP genotyping. An aliquot of 20 µl (a total of 200 ng) of diluted gDNA of each sample was used for target probe preparation and genotyping using high-resolution Affymetrix custom array of 40894 SNP chip. The assays were performed on Gene Titan platform; the high throughput automated working station. Microarray tiled with probes specific to a genomic position of interest. Amplified total genomic DNA was fragmented it and hybridized to the array. Hybridize solution probes (9 mer) was paired a specific “hapten” to a specific base. DNA ligation was used to covalently bind only the correct base followed by washing, staining, fixing and scanning. Hybridization to the Bead Chip and imaging of the arrays were performed by the Imperial Life Science (P) Ltd., Gurgaon, Haryana, India.The Affymetrix Gene Titan assay was based on 2 colors for genotyping; one probe for heterozygous locus detection while 2 probes for homozygous locus, by an allele-specific single base extension/ligation step. The data files generated after scans were CEL files. The analysis was performed on Affymetrix Genotype Console (GTC) Software version: 4.1. The samples below of DQC <0.82 and SNP call rate <95% were removed from the analysis and genotyping call was directly exported from the software. For clustering of SNP, we also used GTC software to call as separate homozygous and heterozygous cluster.

### Linkage map construction and QTL analysis

All polymorphic markers including segregation distortion loci were mapped using SDL option (segregation distortion locus mapping) taking inclusive composite interval mapping (ICIM) and interval mapping (IM) implemented in QTL IciMapping V4 (http://www.isbreeding.net). SDL mapping using this software helped in restoration of order and position of the distorted markers on linkage map. This was additionally verified by Distorted Map v.1 software^38^. For identification of main effect of additive and digenic epistatic QTL in each environment and for each trait, the ‘IM-ADD’, ‘ICIM-ADD’ and ‘ICIM-EPI’ functions, respectively, of the software were utilized^36,64^. Logarithm of odds (LOD) score peaks ≥2.5 were used to declare the presence of a putative QTL in a given genomic region. A threshold LOD of 5.0 with probability values for entering variables (PIN) of 0.01 was used to declare significant epistatic-QTLs. The ‘Multi-Environment Trials’ (MET) function of the software was also utilized to determine the consensus positions for the major QTL and identification of additive × environment interaction effect QTLs (AE-QTL). MET- QTLs were considered if they accounted for >5% of the variance.

### Bioinformatics tool to identify functional genes located inside or close to the identified QTLs

Genes directly related to the synthesis of storage proteins of rice grain, viz. glutelin, globulin, prolamin and albumin, were downloaded along with their physical position from Rice Annotation project Database^65^ and Oryzabase^66^. Functionally validated genes related to increase in grain protein content were also downloaded along with their physical positions from the gene information table available in QTL Annotation Rice Online Database^67^. The gene located inside the QTL interval region or within 1.0 Mb or nearly 4 cM (considering average genetic to physical distance of 1 cM = 220 kb in rice) either side of the peak marker position were considered to be associated with grain protein content and were probable causative genes for increased protein content in high protein introgression lines. Functions of the identified Protein QTL-linked genes were further determined using Rice Genome Annotation Project Database^68^ and Rice Annotation project Database^65^. Rice expression database (RED) was searched from IC4R website (http://ic4r.org)^69^ for getting RNA-seq data of important functional genes inside QTLs responsible for enhanced grain protein content and ‘Box-plot’ view was generated to show the expression level at different plant parts. An *in silico* expression profile of functional genes located within the detected QTL regions was performed using the embryo- and endosperm-specific gene expression data generated during seed ripening stage of rice cv. Nipponbare available at RiceXPro database (RXP_0012) (http://ricexpro.dna.affrc.go.jp/)^70^. This experiment included expression data of 36 independent microarray experiments conducted during seed development stage (Supplementary Table 16). The physical locations of SNP markers and robust QTLs in high protein introgression lines were represented using Graphical GenoTyping (GGT 2.0) software^71^.

## Supplementary information

Supplementary Files

## References

[1] Potrykus I (2003). Nutritionally enhanced rice to combat malnutrition disorders of the poor. Nutrition Rev..

[2] Fitzgerald MA, McCouch SR, Hall RD (2009). Not just a grain of rice: the quest for quality. Trends in Plant Sci..

[3] Tan YF (2001). Mapping quantitative trait loci for milling quality, protein content and color characteristics of rice using a recombinant inbred line population derived from an elite rice hybrid. Theor. Appl. Genet..

[4] Aluko G (2004). QTL mapping of grain quality traits from the interspecific cross *Oryza sativa* × *O*. *glaberrima*. Theor. Appl. Genet..

[5] Wang LQ (2008). The QTL controlling amino acid content in grains of rice (*Oryza sativa*) are co-localized with the regions involved in the amino acid metabolism pathway. Mol. Breed..

[6] Zhang W (2008). QTL mapping for crude protein and protein fraction contents in rice (*Oryza sativa* L.). J. Cereal Sci..

[7] Qin Y, Kim SM, Sohn JK (2009). QTL analysis of protein content in double-haploid lines of rice. Korean J. Crop Sci..

[8] Yu YH (2009). Genetic relationship between grain yield and the contents of protein and fat in a recombinant inbred population of rice. J. Cereal Sci..

[9] Zhong M (2011). Identification of QTL affecting protein and amino acid contents in rice. Rice Sci..

[10] Lee, G. H., Yun, B. W. & Kim, K. M. Analysis of QTLs associated with the rice quality related gene by double haploid populations. *Int*. *J*. *Genomics*. Article ID 781832 (2014). 10.1155/2014/781832 PMC4247976 25478566

[11] Yun BW, Kim MG, Handoyo T, Kim KM (2014). Analysis of rice grain quality associated quantitative trait loci by using genetic mapping. Am. J. Plant Sci..

[12] Yang, Y. *et al*. Identification of quantitative trait loci responsible for rice grain protein content using chromosome segment substitution lines and fine mapping of *qPC-1* in rice (*Oryza sativa* L.). *Mol*. *Breed*. **35**, 10.1007/s11032-015-0328-z (2015)

[13] Wang, X. *et al*. Genome-wide and gene-based association mapping for rice eating and cooking characteristics and protein content. *Sci*. *Rep*. **7**, 10.1038/s41598-017-17347-5 (2017*)*. 10.1038/s41598-017-17347-5 PMC5722854 29222496

[14] Shi CH, Ge GK, Wu JG, Ye J, Wu P (2006). The dynamic gene expression from different genetic systems for protein and lysine contents of indica rice. Genetica.

[15] Mahmoud AA, Sukumar S, Krishnan HB (2008). Interspecific rice hybrid of *Oryza sativa* × *Oryza nivara* reveals a significant increase in seed protein content. J. Agri. Food Chem..

[16] Thomson MJ (2012). High-throughput single nucleotide polymorphism genotyping for breeding applications in rice using the BeadXpress platform. Mol. Breed..

[17] Yu H, Xie W, Li J, Zhou. F, Zhang Q (2013). A whole-genome SNP array (RICE6K) for genomic breeding in rice. Plant Biotechnol. J..

[18] Thomson J (2017). Large-scale deployment of a rice 6 K SNP array for genetics and breeding applications. Rice.

[19] Chen H (2014). A high density SNP genotyping array for rice biology and molecular breeding. Mol. Plant..

[20] McCouch SR (2010). Development of genome-wide SNP assays for rice. Breed. Sci..

[21] Singh, N. *et al*. Single-copy gene based 50 K SNP chip for genetic studies and molecular breeding in rice. *Sci*. *Rep*. **5**, 10.1038/srep11600 (2015). 10.1038/srep11600 PMC4481378 26111882

[22] Shao, Y. *et al*. Infrared spectroscopy and chemometrics for the starch and protein prediction in irradiated rice. *Food Chem*. **126**, 10.1016/j.foodchem.2010.11.166**(**2011). 10.1016/j.foodchem.2010.11.166 25213968

[23] Xie LH (2014). Optimisation of near-infrared reflectance model in measuring protein and amylose content of rice flour. Food Chem..

[24] Bagchi, T. B. *et al*. Development of NIRS models to predict protein and amylose content of brown rice and proximate compositions rice bran. *Food Chem*. **191**, 10.1016/j.foodchem.2015.05.038**(**2015). 10.1016/j.foodchem.2015.05.038 26258697

[25] Chattopadhyay, K., *et al*. Development of recombinant high yielding lines with improved protein content in rice (*Oryza sativa* L.). *J*. *Agric*. *Sci*., *Cambridge*, 10.1017/S0021859618000230 (2018).

[26] Chattopadhyay K, Das A, Das SP (2011). Grain protein content and genetic diversity of rice in north eastern India. Oryza.

[27] Septiningsih EM, Trijatmiko KR, Moeljopawiro S, McCook SR (2003). Identification of quantitative trait loci for grain quality in an advanced backcross population derived from the *Oryza sativa* variety IR 64 and the wild relative *O*. *rufipogon*. Theor. Appl. Genet..

[28] Tanksley SD, Nelson JC (1996). Advanced backcross QTL analysis: A method for the simultaneous discovery and transfer of valuable QTLs from unadapted germplasm into elite breeding lines. Theor. Appl. Genet..

[29] Bernacchi D, Beck-Bunn T, Eshed Y, Eshed SD (1998). Advanced backcross QTL analysis in tomato. I. Identification of QTLs for traits of agronomic importance from. Lycopersicon hirsutum. Theor. Appl. Genet..

[30] Lu, H. *et al*. QTL-seq identifies an early flowering QTL located near FloweringLocus T in cucumber. T*heorAppl Genet*. *2* **17****(****7**), 10.1007/s00122-014-2313-z (2014). 10.1007/s00122-014-2313-z 24845123

[31] Chai, L *et al*. Advanced backcross QTL analysis for the whole plant growth duration salt tolerance in rice (*Oryza sativa* L.). *J*. *Integrative Agric*. **13**(8), 10.1016/S2095-3119(13)60575-4 (2014).

[32] Swamy BPM, Kaladhar K, Reddy GA, Viraktamath BC, Sarala N (2014). Mapping and introgression of QTL for yield and related traits in two backcross populations derived from *Oryza sativa* cv. Swarna and two accessions of *O*. nivara. J. Genet..

[33] Nagata, K. *et al*. Advanced backcross QTL analysis reveals complicated genetic control of rice grain shape in a *japonica* × *indica* cross. *Breed*. *Sci*. **65**, 10.1270/jsbbs.65.308 (2015). 10.1270/jsbbs.65.308 PMC4542931 26366113

[34] Zhan, H. & Xu, S. Generalized linear mixed model for segregation distortion analysis. *BMC Genet*. **12**, 10.1186/1471-2156-12-97 (2011). 10.1186/1471-2156-12-97 PMC3748016 22078575

[35] Xu, S. & Hu, Z. Mapping quantitative trait loci using distorted markers. *Int*. *J*. *Plant Genomics* (2009). 10.1155/2009/410825 PMC2825659 20182628

[36] Zhang L, Li H, Wang J (2012). The statistical power of Inclusive Composite Interval Mapping in detecting digenic epistasis showing common F2 segregation ratios. J. Integr. Plant Biol..

[37] Meng L, Li H, Zhang L, Wang J (2015). QTL Ici Mapping: Integrated software for genetic linkage map construction and quantitative trait locus mapping in biparental populations. The Crop J..

[38] Xie SQ, Feng JY, Zhang YM (2014). Linkage group correction using epistatic distorted markers in F2 and backcross populations. Heredity.

[39] Swamy M (2017). Association Mapping of Yield and Yield related Traits under Reproductive Stage Drought Stress in Rice (*Oryza sativa* L.). Rice..

[40] Zhang L (2010). Effects of missing marker and segregation distortion on QTL mapping in F2 populations. Theor. Appl. Genet..

[41] Shanmugavadivel, S. V. *et al*. Mapping quantitative trait loci (QTL) for grain size in rice using a RIL population from Basmati 3 indica cross showing high segregation distortion. *Euphytica*, 10.1007/s10681-013-0964-5 (2013).

[42] Zheng L (2012). Genetic relationship between grain chalkiness, protein content, and paste viscosity properties in a backcross inbred population of rice. J. Cereal Sci..

[43] Li W (2011). QTL Mapping for Wheat Flour Color with Additive, Epistatic, and QTL × Environmental Interaction Effects. Agric. Sci. China.

[44] Conti V (2011). Mapping of main and epistatic effect QTLs associated to grain protein and gluten strength using a RIL population of durum wheat. J. Appl. Genet..

[45] Guo Y, Mu P, Liu J, Lu Y, Li Z (2007). QTL mapping and Q x E interaction of grain cooking and nutrient qualities in rice under upland and lowland environments. J. Genet Genomics.

[46] Zhang W (2008). QTL analysis of pasta quality using a composite microsatellite and SNP map of durum wheat. Theor. Appl. Genet..

[47] Kumar J (2011). Introgression of a major gene for high grain protein content in some Indian bread wheat cultivars. Field Crop Res..

[48] Cai S (2013). Grain protein content variation and its association analysis in barley. BMC Plant Biol..

[49] Peng B (2014). Comparative mapping of chalkiness components in rice using five populations across two environments. BMC Genet..

[50] Wang Y (2015). Origin of worldwide cultivated barley revealed by *NAM-1* gene and grain protein content. Front. Plant Sci..

[51] Fan, C. *et al*. Identification of QTLs controlling grain protein concentration using a high-density SNP and SSR linkage map in barley (*Hordeum vulgare* L.). *BMC Plant Bio*. **17**, 10.1186/s12870-017-1067-6 (2017). 10.1186/s12870-017-1067-6 PMC5504602 28697758

[52] Vishwakarmaa MK (2014). Introgression of the high grain protein gene *Gpc-B1* in an elite wheat variety of Indo-Gangetic Plains through marker assisted backcross breeding. Cur. Plant Bio..

[53] Huang, X. *et al*. Genome-wide association study of flowering time and grain yield traits in a worldwide collection of rice germplasm. *Nat*. *Genet*. **44**(1), 10.1038/ng.1018 (2012). 10.1038/ng.1018 22138690

[54] Ogawa M (1987). Purification of protein body-I of rice seed and its polypeptide composition. Plant Cell Physiol..

[55] Yoshida, S. Forno, D. A., Cock, J. H. & Gomez, K. A. Laboratory manual for physiological studies of rice. 3^rd^ ed., IRRI, Manila, 1−83 (1976).

[56] Ju Z, Hettiarachchy N, Rath N (2001). Extraction, denaturation and hydrophobic properties of rice flour proteins. J. Food Sci..

[57] Lowry OH, Rosebrough NJ, Lewis, Farr. A, Randall RJ (1951). Protein measurement with the Folin Phenol reagent. J. Biol. Chem..

[58] Juliano BO (1971). A simplified assay for milled rice amylose. Cereal Sci. Today..

[59] Juliano, B. O. Criteria and tests for rice grain qualities. Rice chemistry and technology (Ed. Juliano, B. O.), *American Association of Cereal Chemists*, *Inc*, *MN2*, 43–524 (1985).

[60] Wang, L. Q. *et al*. Genetic basis of 17 traits and viscosity parameters characterizing the eating and cooking quality of rice. *Theor*. *Appl*. *Genet*. **115**, 10.1007/s00122-007-0580-7 (2007). 10.1007/s00122-007-0580-7 17593343

[61] Federer, W. T. & Wolfinger, R. D. Gauss and SAS for recovering inter block and inter variety information. *Technical Report Series of the Biometrics Unit*, 14853 (1996).

[62] Wolfinger RD, Federer WT, Cordero-Brana O (1997). Recovering Information in Augmented Designs, Using SAS PROC GLM and PROC MIXED. Agron. J..

[63] Murray MG, Thompson WF (1980). Rapid isolation of high molecular weight plant DNA. Nucleic Acids Res..

[64] Li H, Ribaut JM, Li. Z, Wang J (2008). Inclusive composite interval mapping (ICIM) for digenic epistasis of quantitative traits in biparental populations. Theor. Appl. Genet..

[65] Sakai H (2013). Rice Annotation Project Database (RAP-DB): an integrative and interactive database for rice genomics. Plant Cell Physiol..

[66] Kurata N, Yamazaki Y (2006). *Oryza* base: An integrated biological and genome information database for rice. Plant physiol..

[67] Yamamoto E, Yonemaru JI, Yamamoto T, Yano M (2012). OGRO: The Overview of functionally characterized Genes in Rice online database. Rice.

[68] Kawahara Y (2013). Improvement of the *Oryza sativa* Nipponbare reference genome using next generation sequence and optical map data. Rice.

[69] Xia L (2017). Rice Expression Database (RED): an integrated RNA-Seq-derived gene expression database for rice. J Genet Genomics.

[70] Sato Y (2010). RiceXPro: a platform for monitoring gene expression in japonica rice grown under natural field conditions. Nucleic acids res..

[71] Van Berloo R (2008). GGT 2.0: versatile software for visualization and analysis of genetic data. J. Hered..

