# Supplementary material for: Detection of stable QTLs for grain protein content in rice (Oryza sativa L.) employing high throughput phenotyping and genotyping platforms
Source: Sci Rep. 2019 Mar 1;9:3196. doi: 10.1038/s41598-019-39863-2 (PMC6397320; doi:10.1038/s41598-019-39863-2)
Supplement: Supplementary file 1 — Supplementary Files [file 41598_2019_39863_MOESM1_ESM.docx]

Supplementary Files

**Title: Detection of stable QTLs for grain protein content in rice (*Oryza sativa* L.) employing high throughput phenotyping and genotyping platforms**

KrishnenduChattopadhyay*^1^, Lambodar Behera^1^, ToritBaran Bagchi^1^, ShrusheeShibani Sardar^1^, Nutan Moharana^1^, Niraja Rani Patra^1^, Mridul Chakraborti^1^, Avijit Das^2^, BishnuCharan Marndi^1^, Ananta Sarkar^3^, Ngangkham Umakanta^1^, Koushik Chakraborty^1^, Lotan Kumar Bose^1^, Sutapa Sarkar^1^, Soham Ray^4^ and Srigopal Sharma^1^

*ICAR-National Rice Research Institute, Cuttack, Odisha- 753006; e-mail: [krishnenducrri@gmail.com](mailto:krishnenducrri@gmail.com)

^1^ICAR-National Rice Research Institute, Cuttack, India

^2^ICAR- National Institute of Research on Jute and Allied Fibre Technology, Kolkata, India

^3^ICAR- Central Institute for Women in Agriculture, Bhubaneswar, India

^4^ICAR-Central Research Institute for Jute and Allied Fibres, Kolkata, India

Supplementary Table 1. Descriptive statistics of different parameters in individual environment and over the environment (*Env.1*+*Env.2*)

|  | Environments | MD | PH | PL | PN | GRAIN | PY | GWT | GPC | SGPC |
| --- | --- | --- | --- | --- | --- | --- | --- | --- | --- | --- |
| Mean | *Env.1* | 126.03 | 117.82 | 26.57 | 8.43 | 89.58 | 21.74 | 2.24 | 9.6 | 1.66 |
|  | *Env.2* | 124.64 | 107.31 | 25.54 | 8.3 | 99.29 | 20.03 | 2.13 | 9.89 | 1.7 |
|  | *Env1*+*Env.2* | 125.3 | 112.56 | 25.56 | 8.37 | 94.44 | 20.89 | 2.18 | 9.75 | 1.66 |
| SE(M) | *Env.1* | 3.41 | 1.63 | 1.07 | 1.03 | 1.46 | 0.97 | 0.08 | 0.19 | 0.06 |
|  | *Env.2* | 4.02 | 1.59 | 1.92 | 1.03 | 1.7 | 1.02 | 0.1 | 0.35 | 0.08 |
|  | *Env1*+*Env.2* | 2.18 | 6.11 | 1.22 | 1.45 | 2.85 | 1.12 | 0.13 | 0.85 | 0.1 |
| h^2^ | *Env.1* | 0.9 | 0.81 | 0.56 | 0.84 | 0.88 | 0.68 | 0.65 | 0.73 | 0.65 |
|  | *Env.2* | 0.89 | 0.96 | 0.53 | 0.89 | 0.86 | 0.64 | 0.61 | 0.79 | 0.62 |
|  | *Env1*+*Env.2* | 0.45 | 0.34 | 0.4 | 0.44 | 0.52 | 0.25 | 0.51 | 0.45 | 0.55 |
| GA(M) | *Env.1* | 4.35 | 16.57 | 8.4 | 52.93 | 57.24 | 68.44 | 3.3 | 25.04 | 7.1 |
|  | *Env.2* | 4.19 | 17.1 | 9.1 | 72.3 | 47.65 | 52.84 | 12.59 | 28.45 | 8.48 |
|  | *Env1*+*Env.2* | 1.95 | 6.75 | 7.16 | 29.69 | 6.11 | 17.52 | 10.62 | 4.43 | 12.31 |
| ECV | *Env.1* | 2.71 | 1.38 | 11.58 | 12.36 | 1.64 | 22.95 | 110.91 | 1.97 | 67.26 |
|  | *Env.2* | 3.23 | 1.48 | 12.16 | 12.53 | 1.7 | 23.92 | 106.66 | 3.64 | 68.91 |
|  | *Env1*+*Env.2* | 2.47 | 7.7 | 6.75 | 24.58 | 34.36 | 28.91 | 17.7 | 12.38 | 13.3 |
| GCV | *Env.1* | 2.86 | 8.16 | 7.5 | 28.67 | 27.83 | 34.17 | 13.37 | 12.31 | 15.42 |
|  | *Env.2* | 2.99 | 8.43 | 8.02 | 37.07 | 23.19 | 32.02 | 25.89 | 14.44 | 17.1 |
|  | *Env1*+*Env.2* | 1.69 | 5.59 | 5.5 | 21.76 | 8.77 | 16.87 | 10.27 | 6.41 | 9.97 |
| PCV | *Env.1* | 3.97 | 8.27 | 13.8 | 30.67 | 27.88 | 41.16 | 111.71 | 12.47 | 69.01 |
|  | *Env.2* | 4.41 | 8.56 | 14.57 | 39.13 | 23.25 | 39.97 | 109.71 | 14.89 | 71 |
|  | *Env1*+*Env.2* | 2.99 | 9.51 | 8.71 | 32.83 | 26.89 | 33.47 | 20.47 | 13.61 | 16.62 |

Foot note: PH: plant height, MD: maturity duration, PN: number of panicles/plant, PL: panicle length, GRAIN: number of grains /panicle, GWT: 100 grain weight, PY: plant yield, GPC: grain protein content (%), SGPC: single grain protein content (mg/g)

Supplementary Table 2.Mean, standard error, CV, range, skewness and kurtosis of yield, yield attributing traits, GPC and SGPC of mapping population over the seasons

| Traits | Mean | SE | CV | Range | Skewness | Kurtosis |
| --- | --- | --- | --- | --- | --- | --- |
| PL | 25.56 | 0.11 | 4.95 | 19-33 | 0.295 | 0.754 |
| PN | 8.37 | 0.14 | 7.53 | 3-18 | 0.548 | 0.394 |
| GRAIN | 94.44 | 1.25 | 598.3 | 27.5-189.5 | 0.531 | 0.843 |
| PY | 20.89 | 0.36 | 48.78 | 7.1-69.6 | 1.16 | 5.34 |
| GWT | 2.18 | 0.23 | 0.2 | 1-4.1 | 1.16 | 3.14 |
| GPC | 9.75 | 0.67 | 1.73 | 5.06-14 | 0.057 | 0.5 |
| SGPC | 1.66 | 0.136 | 0.713 | 1.1-2.87 | 0.763 | 0.986 |

Supplementary Table 3.Correlation coefficient matrix of different traits under *Env.1*, *Env.2* and over the environment (*Env.1*+*Env.2*)

| Parameter |  | MD | PH | PL | PN | GRAIN | GWT | GPC | SGPC | PY |
| --- | --- | --- | --- | --- | --- | --- | --- | --- | --- | --- |
| MD | *Env.1*+*Env.2* | 1.000 |  |  |  |  |  |  |  |  |
|  | *Env.1* | 1.000 |  |  |  |  |  |  |  |  |
|  | *Env.2* | 1.000 |  |  |  |  |  |  |  |  |
| PH | *Env.1*+*Env.2* | 0.259 | 1.000 |  |  |  |  |  |  |  |
|  | *Env.1* | 0.255 | 1.000 |  |  |  |  |  |  |  |
|  | *Env.2* | 0.134 | 1.000 |  |  |  |  |  |  |  |
| PL | *Env.1*+*Env.2* | 0.172 | 0.392 | 1.000 |  |  |  |  |  |  |
|  | *Env.1* | 0.178 | 0.306 | 1.000 |  |  |  |  |  |  |
|  | *Env.2* | 0.021 | 0.120 | 1.000 |  |  |  |  |  |  |
| PN | *Env.1*+*Env.2* | 0.198 | 0.083 | 0.021 | 1.000 |  |  |  |  |  |
|  | *Env.1* | 0.201 | 0.119 | 0.156 | 1.000 |  |  |  |  |  |
|  | *Env.2* | 0.197 | 0.055 | -0.100 | 1.000 |  |  |  |  |  |
| GRAIN | *Env.1*+*Env.2* | 0.047 | -0.065 | 0.031 | -0.089 | 1.000 |  |  |  |  |
|  | *Env.1* | 0.064 | -0.038 | 0.094 | 0.011 | 1.000 |  |  |  |  |
|  | *Env.2* | 0.112 | 0.126 | 0.189 | -0.169 | 1.000 |  |  |  |  |
| GWT | *Env.1*+*Env.2* | 0.024 | 0.155 | 0.024 | 0.048 | -0.123 | 1.000 |  |  |  |
|  | *Env.1* | -0.093 | -0.022 | -0.075 | 0.026 | -0.055 | 1.000 |  |  |  |
|  | *Env.2* | 0.050 | 0.192 | -0.020 | 0.054 | -0.137 | 1.000 |  |  |  |
| GPC | *Env.1*+*Env.2* | -0.030 | -0.060 | -0.134 | 0.067 | -0.136 | 0.077 | 1.000 |  |  |
|  | *Env.1* | -0.131 | -0.066 | -0.067 | -0.026 | -0.135 | 0.002 | 1.000 |  |  |
|  | *Env.2* | 0.087 | 0.040 | -0.122 | 0.130 | -0.188 | 0.134 | 1.000 |  |  |
| SGPC | *Env.1*+*Env.2* | 0.013 | -0.013 | -0.078 | 0.133 | -0.183 | 0.109 | 0.489 | 1.000 |  |
|  | *Env.1* | 0.025 | 0.133 | 0.034 | 0.087 | -0.172 | 0.061 | 0.239 | 1.000 |  |
|  | *Env.2* | 0.068 | 0.052 | -0.016 | 0.174 | -0.283 | 0.173 | 0.649 | 1.000 |  |
| PY | *Env.1*+*Env.2* | 0.206 | 0.194 | 0.210 | 0.311 | 0.248 | -0.048 | -0.018 | 0.025 | 1.000 |
|  | *Env.1* | 0.157 | 0.184 | 0.306 | 0.504 | 0.149 | -0.012 | 0.048 | 0.173 | 1.000 |
|  | *Env.2* | 0.225 | 0.119 | 0.023 | 0.151 | 0.444 | -0.104 | -0.055 | -0.073 | 1.000 |

Supplementary Table 4. Path coefficient analysis of yield over the environment (*Env.1*+*Env.2*)

| Paramters | MD | PH | PL | PN | GRAIN | SW | GPC | SGPC |
| --- | --- | --- | --- | --- | --- | --- | --- | --- |
| MD | **0.079** | 0.020 | 0.014 | 0.016 | 0.004 | 0.002 | -0.002 | 0.001 |
| PH | 0.031 | **0.121** | 0.048 | 0.010 | -0.008 | 0.019 | -0.007 | -0.002 |
| PL | 0.024 | 0.055 | **0.141** | 0.003 | 0.004 | 0.003 | -0.019 | -0.011 |
| PN | 0.060 | 0.025 | 0.006 | **0.303** | -0.027 | 0.014 | 0.020 | 0.040 |
| GRAIN | 0.013 | -0.018 | 0.009 | -0.025 | **0.278** | -0.034 | -0.038 | -0.051 |
| GWT | -0.001 | -0.009 | -0.001 | -0.003 | 0.007 | **-0.058** | -0.004 | -0.006 |
| GPC | 0.000 | -0.001 | -0.001 | 0.001 | -0.001 | 0.001 | **0.009** | 0.005 |
| SGPC | 0.001 | -0.001 | -0.004 | 0.007 | -0.009 | 0.005 | 0.024 | **0.049** |
| PY | 0.206 | 0.194 | 0.210 | 0.311 | 0.248 | -0.048 | -0.018 | 0.025 |
| Partial R² | 0.016 | 0.024 | 0.030 | 0.094 | 0.069 | 0.003 | 0.000 | 0.001 |

Foot note: PH: plant height, MD: maturity duration, PN: number of panicles/plant, PL: panicle length, GRAIN: number of grains /panicle, GWT: 100 grain weight, PY: plant yield, GPC: grain protein content (%), SGPC: single grain protein content (mg/g)

Supplementary Table 5.Distribution of SNP markers on the rice chromosomes (between parents)

| Chromosome | No. SNP markers | Non Polymorphic | | Polymorphic | | Polymorphic | | Total | | GAP | |
| --- | --- | --- | --- | --- | --- | --- | --- | --- | --- | --- | --- |
|  |  |  |  | (Homo) | | (Hetero) | | Polymor. | |  |  |
|  |  | No | % | No | % | No | % | No | % | No | % |
| 1 | 8428 | 6530 | 77.48 | 1415 | 16.79 | 164 | 1.95 | 1579 | 18.74 | 88 | 1.04 |
| 2 | 4987 | 4270 | 85.62 | 594 | 11.91 | 59 | 1.18 | 653 | 13.09 | 59 | 1.18 |
| 3 | 5706 | 5133 | 89.96 | 467 | 8.18 | 42 | 0.74 | 509 | 8.92 | 59 | 1.03 |
| 4 | 3133 | 2393 | 76.38 | 631 | 20.14 | 82 | 2.62 | 713 | 22.76 | 25 | 0.80 |
| 5 | 3563 | 3279 | 92.03 | 205 | 5.75 | 28 | 0.79 | 233 | 6.54 | 47 | 1.32 |
| 6 | 4507 | 3487 | 77.37 | 886 | 19.66 | 80 | 1.78 | 966 | 21.43 | 51 | 1.13 |
| 7 | 1992 | 1748 | 87.75 | 199 | 9.99 | 28 | 1.41 | 227 | 11.40 | 15 | 0.75 |
| 8 | 2051 | 1853 | 90.35 | 139 | 6.78 | 30 | 1.46 | 169 | 8.24 | 29 | 1.41 |
| 9 | 1962 | 1579 | 80.48 | 298 | 15.19 | 34 | 1.73 | 332 | 16.92 | 35 | 1.78 |
| 10 | 983 | 791 | 80.47 | 138 | 14.04 | 30 | 3.05 | 168 | 17.09 | 19 | 1.93 |
| 11 | 1998 | 1576 | 78.88 | 298 | 14.91 | 67 | 3.35 | 365 | 18.27 | 39 | 1.95 |
| 12 | 1584 | 1286 | 81.19 | 222 | 14.02 | 36 | 2.27 | 258 | 16.29 | 40 | 2.53 |
| Total | 40894 | 33925 |  | 5492 |  | 680 |  | 6172 |  | 506 |  |
| Mean | 3407.83 | 2827.08 | 82.96 | 457.67 | 13.43 | 56.67 | 1.66 | 514.3 | 15.09 | 42.17 | 1.24 |
| (p>0.01)df=1 |  |  |  | 975 |  |  |  |  |  |  |  |

| **Supplementary Table 6. Main effect additive QTLs detected in *kharif* 2013 (*Env.1*) for yield attributing traits, GPC and SGPC**   \| sl no. \| Train name \| Chromosome \| Position (cM) \| *QTLs names* \| LeftMarker \| RightMarker \| LOD \| PVE(%) \| Add \| \| --- \| --- \| --- \| --- \| --- \| --- \| --- \| --- \| --- \| --- \| \| 1 \| PL-k \| 8 \| 103 \| *qPL8.1* \| Affx-93223298 \| Affx-93231251 \| 2.7241 \| 8.3259 \| 0.9151 \| \| 2 \| PN-k \| 1 \| 203 \| *qPN1.1* \| Affx-93252632 \| Affx-93252370 \| 2.7421 \| 20.3784 \| 4.2575 \| \| 3 \| PN-k \| 2 \| 8 \| *qPN2.1* \| Affx-93252975 \| Affx-93252552 \| 2.6431 \| 32.7865 \| 2.3221 \| \| 4 \| PN-k \| 2 \| 109 \| *qPN2.2* \| Affx-93226121 \| Affx-93229311 \| 3.0634 \| 32.5301 \| 3.5294 \| \| 5 \| PN-k \| 4 \| 155 \| *qPN4.1* \| Affx-93223455 \| Affx-93259185 \| 3.3891 \| 29.3483 \| 2.9703 \| \| 6 \| PN-k \| 5 \| 205 \| *qPN5.1* \| Affx-93245157 \| Affx-93225592 \| 2.6688 \| 27.9525 \| 3.7464 \| \| 7 \| PN-k \| 5 \| 210 \| *qPN5.2* \| Affx-93251677 \| Affx-93247673 \| 2.8336 \| 22.6275 \| 2.2819 \| \| 8 \| PN-k \| 9 \| 23 \| *qPN9.1* \| Affx-93236164 \| Affx-93228343 \| 5.0542 \| 25.5075 \| 4.0481 \| \| 9 \| PN-k \| 10 \| 19 \| *qPN10.1* \| Affx-93223893 \| Affx-93250665 \| 2.5179 \| 25.1375 \| 3.9673 \| \| 10 \| PN-k \| 11 \| 7 \| *qPN11.1* \| Affx-93251325 \| Affx-93212639 \| 2.8314 \| 23.6868 \| 3.5459 \| \| 11 \| PN-k \| 11 \| 34 \| *qPN11.2* \| Affx-93244240 \| Affx-93261407 \| 3.4871 \| 18.5442 \| 3.7028 \| \| 12 \| PN-k \| 11 \| 44 \| *qPN11.3* \| Affx-93215730 \| Affx-93231700 \| 4.6682 \| 28.8293 \| 3.5782 \| \| 13 \| PN-k \| 12 \| 68 \| *qPN12.1* \| Affx-93242978 \| Affx-93238601 \| 4.1086 \| 23.0237 \| 4.3883 \| \| 14 \| PN-k \| 12 \| 76 \| *qPN12.2* \| Affx-93257146 \| Affx-93240174 \| 2.9498 \| 28.6107 \| 3.8491 \| \| 15 \| GRAIN-k \| 10 \| 38 \| *qGRAIN10.1* \| Affx-93261203 \| Affx-93222453 \| 4.3683 \| 12.0398 \| 18.207 \| \| 16 \| GPC-k \| 1 \| 10 \| *qGPC1.1* \| Affx-93237905 \| Affx-93229368 \| 3.1269 \| 12.1765 \| -0.468 \| \| 17 \| SGPC-k \| 1 \| 10 \| *qSGPC1.1* \| Affx-93237905 \| Affx-93229368 \| 2.8974 \| 10.3696 \| -0.083 \| \| 18 \| SGPC-k \| 2 \| 256 \| *qSGPC2.1* \| Affx-93260438 \| Affx-93236905 \| 3.3156 \| 6.7025 \| 0.0586 \| \| 19 \| SGPC-k \| 7 \| 36 \| *qSGPC7.1* \| Affx-93225742 \| Affx-93256949 \| 3.5095 \| 7.6782 \| 0.0671 \| \| 20 \| SGPC-k \| 11 \| 19 \| *qSGPC11.1* \| Affx-93232878 \| Affx-93212320 \| 2.8732 \| 6.4237 \| 0.0759 \| | | | | | | | | | | |  |  |  |  |  |  |  |  |  |  |  |  |
| --- | --- | --- | --- | --- | --- | --- | --- | --- | --- | --- | --- | --- | --- | --- | --- | --- | --- | --- | --- | --- | --- | --- | --- | --- | --- | --- | --- | --- | --- | --- | --- | --- | --- | --- | --- | --- | --- | --- | --- | --- | --- | --- | --- | --- | --- | --- | --- | --- | --- | --- | --- | --- | --- | --- | --- | --- | --- | --- | --- | --- | --- | --- | --- | --- | --- | --- | --- | --- | --- | --- | --- | --- | --- | --- | --- | --- | --- | --- | --- | --- | --- | --- | --- | --- | --- | --- | --- | --- | --- | --- | --- | --- | --- | --- | --- | --- | --- | --- | --- | --- | --- | --- | --- | --- | --- | --- | --- | --- | --- | --- | --- | --- | --- | --- | --- | --- | --- | --- | --- | --- | --- | --- | --- | --- | --- | --- | --- | --- | --- | --- | --- | --- | --- | --- | --- | --- | --- | --- | --- | --- | --- | --- | --- | --- | --- | --- | --- | --- | --- | --- | --- | --- | --- | --- | --- | --- | --- | --- | --- | --- | --- | --- | --- | --- | --- | --- | --- | --- | --- | --- | --- | --- | --- | --- | --- | --- | --- | --- | --- | --- | --- | --- | --- | --- | --- | --- | --- | --- | --- | --- | --- | --- | --- | --- | --- | --- | --- | --- | --- | --- | --- | --- | --- | --- | --- | --- | --- | --- | --- | --- | --- | --- | --- | --- | --- | --- | --- | --- | --- | --- | --- | --- | --- | --- | --- | --- | --- | --- | --- | --- | --- | --- |
|  |  |  |  |  |  |  |  |  |  |  |  |  |  |  |  |  |  |  |  |  |  |  |
|  |  |  |  |  |  |  |  |  |  |  |  |  |  |  |  |  |  |  |  |  |  |  |

Foot note: PN: number of panicles/plant, PL: panicle length, GRAIN: number of grains /panicle, GPC: grain protein content (%), SGPC: single grain protein content (mg/g)

| **Supplementary Table 7. Main effect additive QTLs detected in *rabi* season 2014 (*Env. 2*) for yield attributing traits, GPC and SGPC** |
| --- |
| \| Sl. No. \| Trait name \| Chromosome \| Position (cM) \| *QTL name* \| LeftMarker \| RightMarker \| LOD \| PVE(%) \| Add \| \| --- \| --- \| --- \| --- \| --- \| --- \| --- \| --- \| --- \| --- \| \| 1 \| PL-r \| 1 \| 88 \| *qPL1.1* \| Affx-93244874 \| Affx-93250440 \| 3.0296 \| 6.1311 \| -0.9346 \| \| 2 \| PL-r \| 6 \| 314 \| *qPL6.1* \| Affx-93241399 \| Affx-93229697 \| 8.2986 \| 24.6735 \| -0.9841 \| \| 3 \| PL-r \| 7 \| 54 \| *qPL7.1* \| Affx-93224300 \| Affx-93245688 \| 2.694 \| 12.3212 \| 2.2749 \| \| 4 \| GRAIN-r \| 1 \| 199 \| *qGRAIN1.1* \| Affx-93223540 \| Affx-93221880 \| 2.7402 \| 7.7448 \| 10.7935 \| \| 5 \| GRAIN-r \| 2 \| 109 \| *qGRAIN2.1* \| Affx-93226121 \| Affx-93229311 \| 2.6213 \| 12.1516 \| 19.4459 \| \| 6 \| GPC-r \| 1 \| 10 \| *qGPC1.1* \| Affx-93237905 \| Affx-93229368 \| 4.017 \| 13.8505 \| -0.5806 \| \| 7 \| GPC-r \| 2 \| 170 \| *qGPC2.1* \| Affx-93221488 \| Affx-93245529 \| 3.1857 \| 17.3537 \| 0.9234 \| \| 8 \| SGPC-r \| 1 \| 144 \| *qSGPC1.2* \| Affx-93230672 \| Affx-93212941 \| 3.3089 \| 18.4627 \| 0.4922 \| \| 9 \| SGPC-r \| 1 \| 254 \| *qSGPC1.3* \| Affx-93228332 \| Affx-93233227 \| 4.0695 \| 16.4014 \| 0.481 \| \| 10 \| SGPC-r \| 1 \| 308 \| *qSGPC1.4* \| Affx-93256957 \| Affx-93252910 \| 3.4864 \| 7.5822 \| 0.0851 \| \| 11 \| SGPC-r \| 2 \| 256 \| *qSGPC2.1* \| Affx-93228464 \| Affx-93256429 \| 3.5278 \| 14.6356 \| 0.5401 \| \| 12 \| SGPC-r \| 3 \| 131 \| *qSGPC3.1* \| Affx-93253793 \| Affx-93260929 \| 4.1154 \| 14.6527 \| 0.5416 \| \| 13 \| SGPC-r \| 7 \| 36 \| *qSGPC7.1* \| Affx-93225742 \| Affx-93256949 \| 3.3281 \| 7.813 \| 0.091 \| \| 14 \| SGPC-r \| 7 \| 47 \| *qSGPC7.2* \| Affx-93258871 \| Affx-93248651 \| 2.6673 \| 17.9426 \| 0.4008 \| \| 15 \| SGPC-r \| 8 \| 15 \| *qSGPC8.1* \| Affx-93222446 \| Affx-93215426 \| 3.5648 \| 14.5859 \| 0.4761 \| \| 16 \| SGPC-r \| 8 \| 69 \| *qSGPC8.2* \| Affx-93259293 \| Affx-93258892 \| 4.5477 \| 23.5471 \| 0.3364 \| \| 17 \| SGPC-r \| 12 \| 77 \| *qSGPC12.1* \| Affx-93257146 \| Affx-93240174 \| 2.966 \| 14.486 \| 0.5305 \| |

Foot note: PN: number of panicles/plant, PL: panicle length, GRAIN: number of grains /panicle, GPC: grain protein content (%), SGPC: single grain protein content (mg/g)

**Supplementary Table 8. QTLs for grain protein content (GPC) and single grain protein content (SGPC) detected by simple interval mapping**

| Trait Name | *Environment* | Chromosome | Position (cM) | Left marker | RightMarker | LOD | PVE(%) | Add | Similarity with QTLs identified by ICIM |
| --- | --- | --- | --- | --- | --- | --- | --- | --- | --- |
| GPC | *Env-1* | 1 | 7 | Affx-93237905 | Affx-93229368 | 3.1275 | 12.1131 | -0.468 | *qGPC1.1* |
|  |  | 3 | 71 | Affx-93224362 | Affx-93247432 | 3.1385 | 19.2853 | -0.6075 |  |
|  |  | 11 | 7 | Affx-93251325 | Affx-93212639 | 2.7942 | 21.7108 | -0.9808 |  |
|  | *Env-2* | 1 | 7 | Affx-93237905 | Affx-93229368 | 3.6135 | 14.3227 | -0.6112 | *qGPC1.1* |
|  | *Env-3* | 1 | 6 | Affx-93237905 | Affx-93229368 | 3.8324 | 13.7879 | -0.4262 | *qGPC1.1* |
|  |  | 3 | 71 | Affx-93224362 | Affx-93247432 | 2.9086 | 16.9676 | -0.499 |  |
|  |  | 6 | 313 | Affx-93241399 | Affx-93229697 | 2.8538 | 6.9143 | 0.2754 |  |
|  |  | 11 | 6 | Affx-93251325 | Affx-93212639 | 3.7121 | 20.2481 | -0.5522 |  |
| SGPC | *Env-1* | 1 | 39 | Affx-93219364 | Affx-93236050 | 3.0894 | 23.1349 | 0.1378 |  |
|  |  | 1 | 118 | Affx-93233764 | Affx-93211938 | 3.3083 | 7.6639 | 0.1707 |  |
|  |  | 2 | 112 | Affx-93237137 | Affx-93232829 | 3.878 | 9.7337 | 0.0752 |  |
|  |  | 2 | 256 | Affx-93260438 | Affx-93236905 | 3.4037 | 8.012 | 0.0643 | *qSGPC2.1* |
|  |  | 3 | 1 | Affx-93213196 | Affx-93253760 | 3.3704 | 8.4116 | 0.0631 |  |
|  |  | 4 | 155 | Affx-93223455 | Affx-93259185 | 3.4582 | 28.7206 | 0.1935 |  |
|  |  | 7 | 36 | Affx-93225742 | Affx-93256949 | 4.3675 | 10.8494 | 0.08 | *qSGPC7.1* |
|  |  | 7 | 45 | Affx-93244194 | Affx-93233337 | 4.577 | 19.0663 | 0.1388 |  |
|  |  | 7 | 55 | Affx-93243672 | Affx-93252192 | 3.1553 | 20.5627 | 0.1772 |  |
|  |  | 11 | 18 | Affx-93232878 | Affx-93212320 | 2.916 | 7.14 | 0.0787 | *qSGPC11.1* |
|  | *Env-2* | 1 | 27 | Affx-93226431 | Affx-93232075 | 3.5358 | 21.4684 | 0.3447 |  |
|  |  | 1 | 38 | Affx-93219364 | Affx-93236050 | 2.9739 | 19.2852 | 0.1691 |  |
|  |  | 1 | 118 | Affx-93233764 | Affx-93211938 | 2.7891 | 6.5016 | 0.2119 |  |
|  |  | 1 | 145 | Affx-93230672 | Affx-93212941 | 2.7874 | 22.2694 | 0.502 | *qSGPC1.2* |
|  |  | 1 | 151 | Affx-93212941 | Affx-93237760 | 2.8089 | 22.1014 | 0.503 |  |
|  |  | 1 | 254 | Affx-93228332 | Affx-93233227 | 5.2562 | 23.0065 | 0.5076 | *qSGPC1.3* |
|  |  | 1 | 366 | Affx-93226573 | Affx-93238009 | 2.8188 | 11.7699 | 0.6729 |  |
|  |  | 1 | 389 | Affx-93219510 | Affx-93243191 | 3.3005 | 18.5492 | 0.2135 |  |
|  |  | 2 | 256 | Affx-93260438 | Affx-93236905 | 3.4314 | 16.0433 | 0.5772 | *qSGPC2.1* |
|  |  | 3 | 131 | Affx-93253793 | Affx-93260929 | 3.9599 | 16.4305 | 0.5829 | *qSGPC3.1* |
|  |  | 7 | 47 | Affx-93258871 | Affx-93248651 | 4.5575 | 25.7557 | 0.4206 | *qSGPC7.2* |
|  |  | 7 | 55 | Affx-93243672 | Affx-93252192 | 3.1733 | 19.3519 | 0.244 |  |
|  |  | 8 | 14 | Affx-93244226 | Affx-93214039 | 3.0194 | 18.3563 | 0.493 |  |
|  |  | 8 | 15 | Affx-93222446 | Affx-93215426 | 3.904 | 18.9205 | 0.4157 | *qSGPC8.1* |
|  |  | 8 | 69 | Affx-93259293 | Affx-93258892 | 4.75 | 27.5273 | 0.3623 | *qSGPC8.2* |
|  |  | 11 | 44 | Affx-93215730 | Affx-93231700 | 3.8152 | 17.1148 | 0.5381 |  |
|  |  | 12 | 77 | Affx-93257146 | Affx-93240174 | 3.5087 | 15.0199 | 0.5628 | *qSGPC12.1* |

**Supplementary Table 9.Epistatic QTLs for single grain protein content (SGPC) in *kharif* 2013 (*Env*. 1)**

| Sl. No. | Ch. 1 | Position (cM) | LeftMarker1 | RightMarker1 | Ch 2 | Position (cM) | LeftMarker2 | RightMarker2 | LOD | PVE(%) | Add1 | Add2 Ad | dbyAdd |
| --- | --- | --- | --- | --- | --- | --- | --- | --- | --- | --- | --- | --- | --- |
| 1 | 3 | 227.9001 | Affx-93222587 | Affx-93251762 | 4 | 38.7 | Affx-93222859 | Affx-93214373 | 3.506 | 3.44452 | 0.0613 | -0.014 | 0.0686 |
| 2 | 3 | 283.8 | Affx-93252421 | Affx-93224503 | 6 | 318.2 | Affx-93241399 | Affx-93229697 | 3.143 | 4.69876 | 0.0449 | -0.038 | 0.0988 |
| 3 | 1 | 382.6998 | Affx-93233072 | Affx-93236651 | 7 | 43 | Affx-93251628 | Affx-93211893 | 3.035 | 3.48716 | 0.026 | 0.0112 | 0.0733 |
| 4 | 1 | 68.8 | Affx-93248733 | Affx-93240263 | 8 | 94.6 | Affx-93213773 | Affx-93216956 | 3.184 | 2.75988 | 0.1457 | 0.1055 | 0.1416 |
| 5 | 2 | 202.1001 | Affx-93225663 | Affx-93231901 | 8 | 107.5 | Affx-93223298 | Affx-93231251 | 3.856 | 5.40472 | -0.105 | 0.0711 | -0.1375 |
| 6 | 3 | 215.0001 | Affx-93237051 | Affx-93215112 | 8 | 107.5 | Affx-93223298 | Affx-93231251 | 3.762 | 5.55892 | -0.101 | 0.0368 | -0.1369 |
| 7 | 8 | 68.8 | Affx-93259293 | Affx-93258892 | 8 | 107.5 | Affx-93223298 | Affx-93231251 | 3.153 | 7.29748 | 0.0946 | 0.0483 | 0.1512 |
| 8 | 8 | 8.6 | Affx-93249002 | Affx-93253378 | 9 | 0 | Affx-93261661 | Affx-93222243 | 3.228 | 3.07976 | 0.0417 | -0.066 | -0.0847 |
| 9 | 8 | 107.5 | Affx-93223298 | Affx-93231251 | 10 | 8.6 | Affx-93241840 | Affx-93235705 | 3.442 | 5.03052 | 0.0046 | -0.085 | -0.125 |
| 10 | 1 | 255 | Affx-93215866 | Affx-93245594 | 11 | 6 | Affx-93251325 | Affx-93212639 | 4.12 | 5.52 | 0.135 | 0.072 | 0.184 |
| 11 | 8 | 107.5 | Affx-93223298 | Affx-93231251 | 11 | 4.3 | Affx-93245327 | Affx-93254562 | 5.792 | 6.09184 | 0.0387 | -0.119 | -0.1322 |

**Supplementary Table 10.Epistatic QTLs for SGPC in *rabi* season 2014 (*Env*. 2)**

| Sl. No. | Ch. 1 | Position (cM) | LeftMarker1 | RightMarker1 | Ch. 2 | Position (cM) | LeftMarker2 | RightMarker2 | LOD | PVE(%) | Add1 | Add2 | AddbyAdd |
| --- | --- | --- | --- | --- | --- | --- | --- | --- | --- | --- | --- | --- | --- |
| 1 | 1 | 24 | Affx-93244257 | Affx-93242258 | 1 | 255 | Affx-93215866 | Affx-93245594 | 3.98 | 7.28596 | 0.21 | 0.228 | 0.2307 |
| 2 | 1 | 36 | Affx-93249932 | Affx-93211437 | 2 | 162 | Affx-93222239 | Affx-93234831 | 3.95 | 6.9774 | 0.16 | 0.223 | 0.2147 |
| 3 | 3 | 33 | Affx-93219818 | Affx-93250093 | 3 | 36 | Affx-93219818 | Affx-93250093 | 4.75 | 10.35328 | -0.2 | 0.177 | -0.271 |
| 4 | 2 | 162 | Affx-93222239 | Affx-93234831 | 3 | 225 | Affx-93251627 | Affx-93241206 | 4.25 | 6.30776 | 0.3 | 0.25 | 0.2524 |
| 5 | 1 | 255 | Affx-93215866 | Affx-93245594 | 3 | 294 | Affx-93213046 | Affx-93222916 | 5.38 | 7.683 | 0.23 | 0.204 | 0.2492 |
| 6 | 1 | 78 | Affx-93212026 | Affx-93243025 | 4 | 150 | Affx-93248936 | Affx-93223314 | 5.02 | 6.15724 | -0.27 | 0.218 | -0.311 |
| 7 | 2 | 186 | Affx-93250538 | Affx-93259944 | 4 | 150 | Affx-93248936 | Affx-93223314 | 3.61 | 5.86684 | -0.29 | 0.256 | -0.288 |
| 8 | 3 | 291 | Affx-93231419 | Affx-93229667 | 4 | 150 | Affx-93248936 | Affx-93223314 | 4.59 | 7.03472 | -0.27 | 0.257 | -0.321 |
| 9 | 4 | 87 | Affx-93227782 | Affx-93259164 | 4 | 150 | Affx-93248936 | Affx-93223314 | 4.06 | 6.01012 | -0.27 | 0.264 | -0.29 |
| 10 | 4 | 150 | Affx-93248936 | Affx-93223314 | 5 | 153 | Affx-93234760 | Affx-93216262 | 3.64 | 5.96104 | 0.24 | 0.284 | 0.2938 |
| 11 | 2 | 162 | Affx-93222239 | Affx-93234831 | 5 | 219 | Affx-93261214 | Affx-93255585 | 4.22 | 6.48128 | 0.31 | 0.226 | 0.2723 |
| 12 | 3 | 225 | Affx-93251627 | Affx-93241206 | 5 | 219 | Affx-93261214 | Affx-93255585 | 6 | 6.19416 | 0.29 | 0.249 | 0.2957 |
| 13 | 1 | 255 | Affx-93215866 | Affx-93245594 | 5 | 222 | Affx-93255585 | Affx-93230954 | 5.81 | 6.07784 | 0.29 | 0.249 | 0.2788 |
| 14 | 5 | 201 | Affx-93217831 | Affx-93245157 | 5 | 222 | Affx-93255585 | Affx-93230954 | 5.31 | 6.05728 | 0.28 | 0.256 | 0.2918 |
| 15 | 6 | 84 | Affx-93213563 | Affx-93253878 | 6 | 300 | Affx-93241738 | Affx-93217322 | 4.23 | 4.42932 | 0.25 | 0.298 | 0.3377 |
| 16 | 1 | 255 | Affx-93215866 | Affx-93245594 | 6 | 312 | Affx-93217322 | Affx-93241399 | 3.53 | 7.30392 | 0.16 | -0.16 | -0.198 |
| 17 | 4 | 150 | Affx-93248936 | Affx-93223314 | 6 | 312 | Affx-93217322 | Affx-93241399 | 3.77 | 6.07804 | 0.24 | -0.27 | -0.291 |
| 18 | 2 | 162 | Affx-93222239 | Affx-93234831 | 6 | 375 | Affx-93245988 | Affx-93218170 | 3.69 | 6.17136 | 0.29 | 0.245 | 0.2509 |
| 19 | 3 | 225 | Affx-93251627 | Affx-93241206 | 6 | 375 | Affx-93245988 | Affx-93218170 | 4.28 | 5.87744 | 0.26 | 0.272 | 0.2814 |
| 20 | 5 | 219 | Affx-93261214 | Affx-93255585 | 6 | 375 | Affx-93245988 | Affx-93218170 | 5.43 | 6.20252 | 0.24 | 0.293 | 0.2923 |
| 21 | 1 | 351 | Affx-93226374 | Affx-93246207 | 7 | 69 | Affx-93222542 | Affx-93218122 | 3.47 | 6.53736 | 0.27 | 0.228 | 0.2849 |
| 22 | 2 | 162 | Affx-93222239 | Affx-93234831 | 7 | 69 | Affx-93222542 | Affx-93218122 | 3.44 | 6.89572 | 0.27 | 0.187 | 0.2387 |
| 23 | 3 | 225 | Affx-93251627 | Affx-93241206 | 7 | 69 | Affx-93222542 | Affx-93218122 | 4.37 | 6.43368 | 0.28 | 0.25 | 0.2972 |
| 24 | 4 | 150 | Affx-93248936 | Affx-93223314 | 7 | 69 | Affx-93222542 | Affx-93218122 | 3.6 | 6.44284 | 0.27 | 0.258 | 0.3028 |
| 25 | 5 | 39 | Affx-93225562 | Affx-93217570 | 7 | 69 | Affx-93222542 | Affx-93218122 | 3.38 | 6.32604 | 0.25 | 0.281 | 0.3238 |
| 26 | 3 | 72 | Affx-93224362 | Affx-93247432 | 8 | 0 | Affx-93222065 | Affx-93238952 | 4.05 | 6.3714 | 0.21 | 0.255 | 0.3044 |
| 27 | 2 | 228 | Affx-93240141 | Affx-93232358 | 8 | 36 | Affx-93229466 | Affx-93214369 | 3.45 | 3.38084 | -0.02 | 4E-04 | -0.084 |
| 28 | 5 | 39 | Affx-93225562 | Affx-93217570 | 8 | 66 | Affx-93253555 | Affx-93240422 | 3.75 | 6.06916 | 0.23 | 0.298 | 0.2931 |
| 29 | 8 | 66 | Affx-93253555 | Affx-93240422 | 8 | 69 | Affx-93259293 | Affx-93258892 | 4.66 | 9.64324 | 0.2 | 0.12 | 0.2158 |
| 30 | 4 | 150 | Affx-93248936 | Affx-93223314 | 8 | 81 | Affx-93245325 | Affx-93240526 | 3.78 | 6.04184 | 0.24 | -0.27 | -0.29 |
| 31 | 1 | 255 | Affx-93215866 | Affx-93245594 | 9 | 0 | Affx-93261661 | Affx-93222243 | 5.23 | 7.36484 | 0.21 | -0.21 | -0.23 |
| 32 | 8 | 108 | Affx-93223298 | Affx-93231251 | 9 | 0 | Affx-93261661 | Affx-93222243 | 4.87 | 9.43416 | 0.2 | -0.22 | -0.246 |
| 33 | 4 | 150 | Affx-93248936 | Affx-93223314 | 9 | 30 | Affx-93226913 | Affx-93227730 | 4.34 | 6.92752 | 0.25 | -0.27 | -0.317 |
| 34 | 2 | 192 | Affx-93259944 | Affx-93211604 | 9 | 57 | Affx-93213198 | Affx-93225425 | 3.78 | 7.29648 | 0.22 | 0.239 | 0.2598 |
| 35 | 3 | 210 | Affx-93249286 | Affx-93219763 | 9 | 57 | Affx-93213198 | Affx-93225425 | 5.53 | 6.84356 | 0.23 | 0.261 | 0.242 |
| 36 | 5 | 219 | Affx-93261214 | Affx-93255585 | 9 | 57 | Affx-93213198 | Affx-93225425 | 5.01 | 6.23264 | 0.24 | 0.295 | 0.2857 |
| 37 | 6 | 375 | Affx-93245988 | Affx-93218170 | 9 | 57 | Affx-93213198 | Affx-93225425 | 4.56 | 5.99952 | 0.26 | 0.287 | 0.2586 |
| 38 | 9 | 30 | Affx-93226913 | Affx-93227730 | 9 | 57 | Affx-93213198 | Affx-93225425 | 3.61 | 7.7534 | -0.18 | 0.211 | -0.227 |
| 39 | 4 | 84 | Affx-93216491 | Affx-93225282 | 10 | 3 | Affx-93241840 | Affx-93235705 | 3.27 | 6.85284 | 0.14 | 0.112 | 0.1602 |
| 40 | 3 | 291 | Affx-93231419 | Affx-93229667 | 10 | 18 | Affx-93223893 | Affx-93250665 | 3.81 | 6.92256 | -0.23 | 0.292 | -0.285 |
| 41 | 5 | 219 | Affx-93261214 | Affx-93255585 | 10 | 18 | Affx-93223893 | Affx-93250665 | 3.9 | 6.155 | 0.24 | 0.297 | 0.2876 |
| 42 | 8 | 69 | Affx-93259293 | Affx-93258892 | 10 | 75 | Affx-93220668 | Affx-93213633 | 4.97 | 9.95848 | 0.09 | -0.15 | -0.255 |
| 43 | 8 | 105 | Affx-93223298 | Affx-93231251 | 11 | 0 | Affx-93212249 | Affx-93243433 | 3.79 | 5.31592 | 0.07 | -0.12 | -0.156 |
| 44 | 1 | 255 | Affx-93215866 | Affx-93245594 | 11 | 6 | Affx-93251325 | Affx-93212639 | 5.69 | 8.41568 | 0.21 | 0.177 | 0.2401 |
| 45 | 3 | 225 | Affx-93251627 | Affx-93241206 | 11 | 6 | Affx-93251325 | Affx-93212639 | 3.79 | 6.55904 | 0.28 | 0.249 | 0.2966 |
| 46 | 4 | 150 | Affx-93248936 | Affx-93223314 | 11 | 6 | Affx-93251325 | Affx-93212639 | 3.71 | 6.66712 | 0.27 | 0.255 | 0.3026 |
| 47 | 5 | 165 | Affx-93223833 | Affx-93258331 | 11 | 6 | Affx-93251325 | Affx-93212639 | 4.29 | 8.0084 | 0.24 | 0.178 | 0.2292 |
| 48 | 6 | 375 | Affx-93245988 | Affx-93218170 | 11 | 6 | Affx-93251325 | Affx-93212639 | 3.3 | 6.54536 | 0.28 | 0.251 | 0.298 |
| 49 | 7 | 45 | Affx-93244194 | Affx-93233337 | 11 | 6 | Affx-93251325 | Affx-93212639 | 3.1 | 6.3002 | 0.15 | 0.16 | 0.221 |
| 50 | 9 | 57 | Affx-93213198 | Affx-93225425 | 11 | 6 | Affx-93251325 | Affx-93212639 | 5.17 | 8.20652 | 0.23 | 0.183 | 0.241 |
| 51 | 1 | 93 | Affx-93252810 | Affx-93222162 | 12 | 9 | Affx-93232773 | Affx-93214513 | 3.81 | 6.47228 | -0.25 | 0.229 | -0.302 |
| 52 | 2 | 186 | Affx-93250538 | Affx-93259944 | 12 | 9 | Affx-93232773 | Affx-93214513 | 3.92 | 6.10188 | -0.29 | 0.237 | -0.293 |
| 53 | 4 | 87 | Affx-93227782 | Affx-93259164 | 12 | 9 | Affx-93232773 | Affx-93214513 | 4.18 | 6.32008 | -0.28 | 0.242 | -0.297 |
| 54 | 6 | 312 | Affx-93217322 | Affx-93241399 | 12 | 9 | Affx-93232773 | Affx-93214513 | 4.2 | 6.46312 | -0.28 | 0.232 | -0.301 |
| 55 | 8 | 81 | Affx-93245325 | Affx-93240526 | 12 | 9 | Affx-93232773 | Affx-93214513 | 4.19 | 6.40084 | -0.28 | 0.232 | -0.3 |
| 56 | 10 | 27 | Affx-93241409 | Affx-93212950 | 12 | 9 | Affx-93232773 | Affx-93214513 | 3.65 | 6.69792 | -0.2 | 0.154 | -0.373 |
| 57 | 3 | 291 | Affx-93231419 | Affx-93229667 | 12 | 12 | Affx-93232773 | Affx-93214513 | 5.19 | 7.1288 | -0.27 | 0.259 | -0.32 |
| 58 | 5 | 219 | Affx-93261214 | Affx-93255585 | 12 | 12 | Affx-93232773 | Affx-93214513 | 4.91 | 6.3044 | 0.27 | 0.266 | 0.315 |
| 59 | 11 | 6 | Affx-93251325 | Affx-93212639 | 12 | 12 | Affx-93232773 | Affx-93214513 | 4.15 | 6.8596 | 0.26 | 0.255 | 0.3176 |
| 60 | 7 | 69 | Affx-93222542 | Affx-93218122 | 12 | 18 | Affx-93214513 | Affx-93253798 | 3.63 | 6.37196 | 0.26 | 0.276 | 0.3048 |
| 61 | 9 | 27 | Affx-93228343 | Affx-93226913 | 12 | 18 | Affx-93214513 | Affx-93253798 | 4.8 | 9.47728 | -0.24 | 0.238 | -0.324 |
| 62 | 12 | 15 | Affx-93214513 | Affx-93253798 | 12 | 72 | Affx-93236988 | Affx-93257146 | 3.43 | 3.0594 | 0.17 | 0.188 | 0.2251 |

| **Supplementary Table 11. Epistatic x environment interaction QTLs for GPC** | | | | | | | | | | | | | | | | | | | | | | |
| --- | --- | --- | --- | --- | --- | --- | --- | --- | --- | --- | --- | --- | --- | --- | --- | --- | --- | --- | --- | --- | --- | --- |
| Chromosome1 | Position (cM) | LeftMarker1 | RightMarker1 | Chromosome2 | Position (cM) | LeftMarker2 | RightMarker2 | LOD | LOD(AA) | LOD(AAbyE) | PVE | PVE(AA) | PVE(AAbyE) | Add1 | Add2 | AddbyAdd | A1byE_01 | A1byE_02 | A2byE_01 | A2byE_02 | AAbyE_01 | AAbyE_02 |
| 1 | 378 | Affx-93257901 | Affx-93221566 | 2 | 90 | Affx-93215717 | Affx-93244986 | 5.1735 | 4.9016 | 0.2719 | 4.9701 | 4.7643 | 0.2058 | -0.6253 | -0.765 | -0.7912 | 0.1544 | -0.1544 | 0.2329 | -0.2329 | 0.1836 | -0.1836 |
| 2 | 156 | Affx-93240031 | Affx-93237540 | 3 | 174 | Affx-93237410 | Affx-93224100 | 5.7032 | 4.8521 | 0.851 | 6.0155 | 5.1844 | 0.8312 | 0.1321 | 0.1302 | 0.3331 | -0.0923 | 0.0923 | 0.0028 | -0.0028 | -0.1378 | 0.1378 |
| 1 | 276 | Affx-93223593 | Affx-93233872 | 3 | 270 | Affx-93244915 | Affx-93221386 | 6.4525 | 6.2654 | 0.1871 | 6.0792 | 5.8868 | 0.1925 | -0.3405 | -0.4483 | -0.5481 | 0.0581 | -0.0581 | 0.0379 | -0.0379 | 0.0955 | -0.0955 |
| 3 | 96 | Affx-93249508 | Affx-93211644 | 4 | 60 | Affx-93219226 | Affx-93223292 | 7.1779 | 6.9129 | 0.265 | 7.2087 | 6.9167 | 0.292 | 0.1521 | 0.2117 | 0.4095 | 0.0162 | -0.0162 | -0.0564 | 0.0564 | -0.0786 | 0.0786 |
| 2 | 240 | Affx-93254521 | Affx-93212050 | 5 | 234 | Affx-93246459 | Affx-93225634 | 5.1246 | 5.0358 | 0.0888 | 5.1105 | 5.0448 | 0.0658 | -0.276 | -0.0587 | -0.4101 | 0.0374 | -0.0374 | -0.029 | 0.029 | 0.0534 | -0.0534 |
| 3 | 288 | Affx-93245421 | Affx-93231419 | 6 | 336 | Affx-93253277 | Affx-93252098 | 5.0378 | 4.8058 | 0.2321 | 5.4047 | 5.1785 | 0.2262 | 0.3322 | -0.1361 | 0.4349 | -0.0282 | 0.0282 | 0.0552 | -0.0552 | -0.0908 | 0.0908 |

| **Suplementary Table 12. Epistatic x environment interaction QTLs for SGPC** | | | | | | | | | | | | | | | | | | | | | | |
| --- | --- | --- | --- | --- | --- | --- | --- | --- | --- | --- | --- | --- | --- | --- | --- | --- | --- | --- | --- | --- | --- | --- |
| Chomosome1 | Position (cM) | LeftMarker1 | RightMarker1 | Chromosome2 | Position (cM) | LeftMarker2 | RightMarker2 | LOD | LOD(AA) | LOD(AAbyE) | PVE | PVE(AA) | PVE(AAbyE) | Add1 | Add2 | AddbyAdd | A1byE_01 | A1byE_02 | A2byE_01 | A2byE_02 | AAbyE_01 | AAbyE_02 |
| 1 | 255 | Affx-93215866 | Affx-93245594 | 2 | 186 | Affx-93250538 | Affx-93259944 | 6.7048 | 6.3863 | 0.3185 | 7.5617 | 7.3554 | 0.2063 | 0.072 | -0.0852 | -0.1051 | -0.0165 | 0.0165 | 0.0169 | -0.0169 | 0.0189 | -0.0189 |
| 3 | 33 | Affx-93219818 | Affx-93250093 | 3 | 36 | Affx-93219818 | Affx-93250093 | 5.3005 | 4.4401 | 0.8604 | 3.2168 | 3.2168 | 0 | -0.0902 | 0.0888 | -0.1109 | 0.0106 | -0.0106 | -0.0163 | 0.0163 | 0.022 | -0.022 |
| 1 | 255 | Affx-93215866 | Affx-93245594 | 3 | 294 | Affx-93213046 | Affx-93222916 | 8.2263 | 7.1939 | 1.0323 | 11.3109 | 10.8379 | 0.4731 | 0.141 | 0.1188 | 0.1592 | -0.0344 | 0.0344 | -0.0312 | 0.0312 | -0.0342 | 0.0342 |
| 1 | 36 | Affx-93249932 | Affx-93211437 | 4 | 150 | Affx-93248936 | Affx-93223314 | 6.3665 | 5.2439 | 1.1226 | 4.214 | 3.7169 | 0.4971 | 0.1665 | 0.1512 | 0.1876 | -0.0631 | 0.0631 | -0.077 | 0.077 | -0.0682 | 0.0682 |
| 2 | 186 | Affx-93250538 | Affx-93259944 | 4 | 150 | Affx-93248936 | Affx-93223314 | 5.5802 | 4.6163 | 0.9639 | 3.1837 | 2.8446 | 0.3392 | -0.1783 | 0.1412 | -0.175 | 0.0669 | -0.0669 | -0.0705 | 0.0705 | 0.0658 | -0.0658 |
| 3 | 291 | Affx-93231419 | Affx-93229667 | 4 | 150 | Affx-93248936 | Affx-93223314 | 7.8142 | 6.667 | 1.1471 | 6.6707 | 5.8432 | 0.8275 | -0.1697 | 0.1518 | -0.2104 | 0.0694 | -0.0694 | -0.0764 | 0.0764 | 0.0757 | -0.0757 |
| 4 | 87 | Affx-93227782 | Affx-93259164 | 4 | 150 | Affx-93248936 | Affx-93223314 | 6.5245 | 5.5037 | 1.0208 | 3.5002 | 3.2349 | 0.2653 | -0.1769 | 0.1514 | -0.1879 | 0.0671 | -0.0671 | -0.0782 | 0.0782 | 0.0693 | -0.0693 |
| 4 | 150 | Affx-93248936 | Affx-93223314 | 5 | 153 | Affx-93234760 | Affx-93216262 | 5.3283 | 4.5846 | 0.7436 | 3.5255 | 3.1834 | 0.3421 | 0.1078 | 0.1635 | 0.1687 | -0.0568 | 0.0568 | -0.055 | 0.055 | -0.0546 | 0.0546 |
| 3 | 225 | Affx-93251627 | Affx-93241206 | 5 | 219 | Affx-93261214 | Affx-93255585 | 7.8555 | 6.0778 | 1.7777 | 8.3328 | 7.0631 | 1.2696 | 0.1801 | 0.1419 | 0.1799 | -0.0737 | 0.0737 | -0.0703 | 0.0703 | -0.0765 | 0.0765 |
| 1 | 255 | Affx-93215866 | Affx-93245594 | 5 | 222 | Affx-93255585 | Affx-93230954 | 8.1322 | 6.4367 | 1.6956 | 7.4787 | 6.4227 | 1.056 | 0.185 | 0.1464 | 0.1753 | -0.0772 | 0.0772 | -0.0727 | 0.0727 | -0.075 | 0.075 |
| 5 | 201 | Affx-93217831 | Affx-93245157 | 5 | 222 | Affx-93255585 | Affx-93230954 | 6.9956 | 5.3927 | 1.6029 | 5.5529 | 5.5529 | 0 | 0.1733 | 0.1691 | 0.1968 | -0.0841 | 0.0841 | -0.078 | 0.078 | -0.0748 | 0.0748 |
| 6 | 255 | Affx-93233846 | Affx-93257088 | 6 | 264 | Affx-93231709 | Affx-93233066 | 6.3354 | 5.6177 | 0.7177 | 5.1682 | 5.1682 | 0 | 0.0501 | -0.0855 | -0.2569 | -0.0179 | 0.0179 | 0.0263 | -0.0263 | 0.0844 | -0.0844 |
| 1 | 255 | Affx-93215866 | Affx-93245594 | 6 | 312 | Affx-93217322 | Affx-93241399 | 5.8819 | 5.6171 | 0.2648 | 6.8904 | 6.7448 | 0.1456 | 0.0689 | -0.073 | -0.1036 | -0.0175 | 0.0175 | 0.0126 | -0.0126 | 0.0177 | -0.0177 |
| 4 | 150 | Affx-93248936 | Affx-93223314 | 6 | 312 | Affx-93217322 | Affx-93241399 | 5.6806 | 4.8472 | 0.8334 | 3.3257 | 3.007 | 0.3188 | 0.1245 | -0.1606 | -0.1713 | -0.0631 | 0.0631 | 0.0565 | -0.0565 | 0.0596 | -0.0596 |
| 5 | 222 | Affx-93255585 | Affx-93230954 | 6 | 375 | Affx-93245988 | Affx-93218170 | 6.4362 | 5.095 | 1.3412 | 6.9244 | 6.0474 | 0.877 | 0.0975 | 0.1372 | 0.1345 | -0.0477 | 0.0477 | -0.0478 | 0.0478 | -0.0503 | 0.0503 |
| 5 | 9 | Affx-93256879 | Affx-93254815 | 7 | 12 | Affx-93255244 | Affx-93234810 | 5.1305 | 4.5731 | 0.5574 | 5.8205 | 5.4766 | 0.3439 | 0.0874 | 0.1051 | 0.123 | -0.0174 | 0.0174 | -0.0301 | 0.0301 | -0.0307 | 0.0307 |
| 4 | 150 | Affx-93248936 | Affx-93223314 | 7 | 69 | Affx-93222542 | Affx-93218122 | 6.0234 | 4.8358 | 1.1876 | 4.4099 | 3.808 | 0.6019 | 0.1662 | 0.171 | 0.1987 | -0.0797 | 0.0797 | -0.0722 | 0.0722 | -0.0754 | 0.0754 |
| 1 | 363 | Affx-93226573 | Affx-93238009 | 8 | 69 | Affx-93259293 | Affx-93258892 | 6.3604 | 6.004 | 0.3565 | 6.6171 | 6.5108 | 0.1063 | -0.1167 | 0.0611 | -0.1637 | 0.0315 | -0.0315 | -0.0215 | 0.0215 | 0.0289 | -0.0289 |
| 2 | 171 | Affx-93249375 | Affx-93214388 | 8 | 69 | Affx-93259293 | Affx-93258892 | 7.7614 | 7.4357 | 0.3257 | 14.2856 | 13.989 | 0.2966 | -0.17 | 0.0405 | -0.2009 | 0.0263 | -0.0263 | -0.0152 | 0.0152 | 0.0307 | -0.0307 |
| 3 | 288 | Affx-93245421 | Affx-93231419 | 8 | 69 | Affx-93259293 | Affx-93258892 | 9.5702 | 8.9788 | 0.5913 | 10.8232 | 10.1478 | 0.6754 | -0.1374 | 0.1503 | -0.1578 | 0.0418 | -0.0418 | -0.0431 | 0.0431 | 0.0387 | -0.0387 |
| 4 | 57 | Affx-93260627 | Affx-93245124 | 8 | 69 | Affx-93259293 | Affx-93258892 | 6.9165 | 6.7214 | 0.195 | 4.4343 | 4.3437 | 0.0906 | -0.1088 | 0.114 | -0.1336 | 0.0249 | -0.0249 | -0.0294 | 0.0294 | 0.0282 | -0.0282 |
| 5 | 234 | Affx-93246459 | Affx-93225634 | 8 | 69 | Affx-93259293 | Affx-93258892 | 7.9996 | 6.9136 | 1.086 | 10.8473 | 10.0679 | 0.7795 | 0.1974 | 0.1389 | 0.2019 | -0.0547 | 0.0547 | -0.0513 | 0.0513 | -0.0551 | 0.0551 |
| 6 | 84 | Affx-93213563 | Affx-93253878 | 8 | 69 | Affx-93259293 | Affx-93258892 | 10.9016 | 6.8841 | 4.0174 | 2.2816 | 1.6084 | 0.6732 | 0.0608 | 0.2922 | 0.1239 | -0.0685 | 0.0685 | -0.1103 | 0.1103 | -0.079 | 0.079 |
| 7 | 69 | Affx-93222542 | Affx-93218122 | 8 | 69 | Affx-93259293 | Affx-93258892 | 5.9782 | 4.4471 | 1.5311 | 5.7137 | 5.1125 | 0.6012 | 0.1456 | 0.1379 | 0.1766 | -0.0609 | 0.0609 | -0.0631 | 0.0631 | -0.065 | 0.065 |
| 8 | 36 | Affx-93229466 | Affx-93214369 | 8 | 69 | Affx-93259293 | Affx-93258892 | 9.6905 | 8.5951 | 1.0955 | 18.9396 | 18.1034 | 0.8362 | 0.2002 | 0.0209 | 0.2175 | -0.0541 | 0.0541 | -0.0033 | 0.0033 | -0.0469 | 0.0469 |
| 1 | 255 | Affx-93215866 | Affx-93245594 | 9 | 0 | Affx-93261661 | Affx-93222243 | 7.3055 | 6.3098 | 0.9957 | 7.1264 | 6.7108 | 0.4156 | 0.1191 | -0.1147 | -0.1293 | -0.0323 | 0.0323 | 0.03 | -0.03 | 0.0355 | -0.0355 |
| 4 | 150 | Affx-93248936 | Affx-93223314 | 9 | 30 | Affx-93226913 | Affx-93227730 | 6.9668 | 5.9502 | 1.0166 | 5.4797 | 4.8717 | 0.6081 | 0.1378 | -0.165 | -0.1947 | -0.0697 | 0.0697 | 0.0623 | -0.0623 | 0.0662 | -0.0662 |
| 3 | 294 | Affx-93213046 | Affx-93222916 | 9 | 57 | Affx-93213198 | Affx-93225425 | 8.358 | 7.1828 | 1.1752 | 9.3235 | 8.7173 | 0.6062 | 0.1455 | 0.1739 | 0.1796 | -0.0484 | 0.0484 | -0.0547 | 0.0547 | -0.0522 | 0.0522 |
| 5 | 219 | Affx-93261214 | Affx-93255585 | 9 | 57 | Affx-93213198 | Affx-93225425 | 6.418 | 5.2253 | 1.1928 | 7.4999 | 6.4529 | 1.0469 | 0.1068 | 0.1511 | 0.1493 | -0.052 | 0.052 | -0.0613 | 0.0613 | -0.0595 | 0.0595 |
| 6 | 375 | Affx-93245988 | Affx-93218170 | 9 | 57 | Affx-93213198 | Affx-93225425 | 5.7358 | 4.3828 | 1.353 | 3.4288 | 2.9397 | 0.4891 | 0.1422 | 0.1491 | 0.1333 | -0.058 | 0.058 | -0.0663 | 0.0663 | -0.0602 | 0.0602 |
| 8 | 69 | Affx-93259293 | Affx-93258892 | 9 | 63 | Affx-93238893 | Affx-93235378 | 8.4447 | 7.9964 | 0.4483 | 8.1966 | 7.959 | 0.2376 | 0.061 | -0.1402 | -0.177 | -0.0197 | 0.0197 | 0.029 | -0.029 | 0.0332 | -0.0332 |
| 5 | 219 | Affx-93261214 | Affx-93255585 | 10 | 18 | Affx-93223893 | Affx-93250665 | 5.041 | 3.9225 | 1.1185 | 4.1691 | 3.7313 | 0.4378 | 0.1443 | 0.1583 | 0.1724 | -0.0582 | 0.0582 | -0.0609 | 0.0609 | -0.0591 | 0.0591 |
| 8 | 69 | Affx-93259293 | Affx-93258892 | 10 | 54 | Affx-93261516 | Affx-93258720 | 5.2026 | 5.088 | 0.1146 | 2.4096 | 2.3787 | 0.0309 | 0.06 | -0.1311 | -0.1347 | -0.021 | 0.021 | 0.027 | -0.027 | 0.0227 | -0.0227 |
| 3 | 183 | Affx-93239455 | Affx-93249777 | 11 | 0 | Affx-93212249 | Affx-93243433 | 5.0208 | 4.5722 | 0.4486 | 4.9659 | 4.5515 | 0.4145 | 0.0144 | -0.0325 | -0.0597 | 0.0004 | -0.0004 | 0.0006 | -0.0006 | 0.0184 | -0.0184 |
| 8 | 105 | Affx-93223298 | Affx-93231251 | 11 | 0 | Affx-93212249 | Affx-93243433 | 9.0215 | 8.9126 | 0.1089 | 11.1308 | 11.0471 | 0.0837 | 0.0452 | -0.1105 | -0.1331 | -0.01 | 0.01 | 0.0018 | -0.0018 | 0.0116 | -0.0116 |
| 1 | 255 | Affx-93215866 | Affx-93245594 | 11 | 6 | Affx-93251325 | Affx-93212639 | 9.2612 | 8.3975 | 0.8637 | 12.6073 | 12.1099 | 0.4974 | 0.1244 | 0.1012 | 0.1507 | -0.0297 | 0.0297 | -0.0243 | 0.0243 | -0.0312 | 0.0312 |
| 4 | 150 | Affx-93248936 | Affx-93223314 | 11 | 6 | Affx-93251325 | Affx-93212639 | 6.063 | 4.916 | 1.1469 | 4.2765 | 3.7471 | 0.5295 | 0.152 | 0.1609 | 0.187 | -0.0727 | 0.0727 | -0.0632 | 0.0632 | -0.0677 | 0.0677 |
| 9 | 57 | Affx-93213198 | Affx-93225425 | 11 | 6 | Affx-93251325 | Affx-93212639 | 7.3473 | 6.1919 | 1.1554 | 9.786 | 9.0629 | 0.7231 | 0.1421 | 0.109 | 0.1495 | -0.0432 | 0.0432 | -0.0356 | 0.0356 | -0.0434 | 0.0434 |
| 3 | 291 | Affx-93231419 | Affx-93229667 | 12 | 9 | Affx-93232773 | Affx-93214513 | 6.8409 | 5.3194 | 1.5215 | 4.4637 | 3.9753 | 0.4883 | -0.1682 | 0.1494 | -0.2056 | 0.0772 | -0.0772 | -0.08 | 0.08 | 0.0823 | -0.0823 |
| 11 | 6 | Affx-93251325 | Affx-93212639 | 12 | 9 | Affx-93232773 | Affx-93214513 | 5.1117 | 3.8484 | 1.2633 | 3.3463 | 2.9893 | 0.357 | 0.1603 | 0.139 | 0.1857 | -0.0626 | 0.0626 | -0.0635 | 0.0635 | -0.0668 | 0.0668 |
| 5 | 222 | Affx-93255585 | Affx-93230954 | 12 | 12 | Affx-93232773 | Affx-93214513 | 6.8091 | 5.4794 | 1.3297 | 5.8524 | 5.0103 | 0.8421 | 0.148 | 0.1292 | 0.1683 | -0.0657 | 0.0657 | -0.0611 | 0.0611 | -0.0629 | 0.0629 |
| 9 | 0 | Affx-93261661 | Affx-93222243 | 12 | 12 | Affx-93232773 | Affx-93214513 | 5.5845 | 4.6102 | 0.9743 | 4.6288 | 4.2107 | 0.4181 | -0.1482 | 0.1211 | -0.15 | 0.0475 | -0.0475 | -0.0517 | 0.0517 | 0.0499 | -0.0499 |
| 2 | 102 | Affx-93241567 | Affx-93213418 | 12 | 15 | Affx-93214513 | Affx-93253798 | 6.4684 | 6.0053 | 0.463 | 6.4321 | 5.9612 | 0.4709 | 0.1251 | 0.0937 | 0.1499 | -0.0361 | 0.0361 | -0.0385 | 0.0385 | -0.0407 | 0.0407 |
| 7 | 9 | Affx-93229571 | Affx-93246611 | 12 | 15 | Affx-93214513 | Affx-93253798 | 5.3385 | 5.0384 | 0.3001 | 5.1958 | 4.9258 | 0.27 | 0.11 | 0.0586 | 0.1276 | -0.0286 | 0.0286 | -0.0278 | 0.0278 | -0.0305 | 0.0305 |
| 6 | 84 | Affx-93213563 | Affx-93253878 | 12 | 18 | Affx-93214513 | Affx-93253798 | 5.7507 | 4.7801 | 0.9706 | 5.8661 | 4.8741 | 0.992 | 0.1417 | 0.2 | 0.2169 | -0.0785 | 0.0785 | -0.0985 | 0.0985 | -0.0915 | 0.0915 |
| 1 | 303 | Affx-93246403 | Affx-93225164 | 12 | 75 | Affx-93257146 | Affx-93240174 | 5.2471 | 4.777 | 0.4701 | 6.0224 | 5.5676 | 0.4549 | 0.1223 | 0.0308 | 0.1361 | -0.0172 | 0.0172 | -0.0225 | 0.0225 | -0.0384 | 0.0384 |
| 8 | 69 | Affx-93259293 | Affx-93258892 | 12 | 75 | Affx-93257146 | Affx-93240174 | 5.8869 | 5.5295 | 0.3574 | 6.4018 | 6.2996 | 0.1023 | -0.0579 | -0.1887 | -0.2412 | 0.0029 | -0.0029 | 0.0266 | -0.0266 | 0.0397 | -0.0397 |
| 12 | 6 | Affx-93248668 | Affx-93255795 | 12 | 75 | Affx-93257146 | Affx-93240174 | 5.6139 | 5.3905 | 0.2234 | 5.8007 | 5.5427 | 0.258 | 0.1231 | 0.0091 | 0.1395 | -0.0135 | 0.0135 | -0.0154 | 0.0154 | -0.0267 | 0.0267 |

Supplementary Table 13. Evaluation of selected high yielding introgression lines (BC_3_F_4_) in Naveen background for GPC (%), single grain protein content (SGPC) (mg/g), grain yield (kg/ha) and protein yield (kg/ha)

|  | |  |  | High GPC donor ARC10075 | Check-1 | Introgression lines in Naveen background | | | | | | |
| --- | --- | --- | --- | --- | --- | --- | --- | --- | --- | --- | --- | --- |
| Trait (season wise) | | | Parameters |  | Naveen | PLN-100 | PLN-116 | PLN-23 | PLN-32 | PLN-37 | PLN-99 | PLN-98 |
|  | *Rabi* 2015 (*Env*. 4) | | GPC (%) | 10.80 | 7.74 | 10.40 | 11.17 | 12.10 | 11.90 | 10.51 | 10.15 | 9.86 |
|  |  |  | SGPC (mg) | 2.40 | 1.56 | 1.98 | 1.68 | 2.15 | 1.85 | 1.93 | 1.99 | 1.94 |
|  | *Kharif*2015 (*Env*.5) | | GPC (%) | 10.88 | 8.13 | 9.47 | 10.43 | 9.72 | 9.47 | 9.79 | 10.45 | 10.25 |
|  |  |  | SGPC (mg) | 2.58 | 1.60 | 2.10 | 2.14 | 2.16 | 2.00 | 2.07 | 2.08 | 2.14 |
|  | Mean | | GPC (%) | 10.84 | 7.93 | 9.93 | 10.80 | 10.91 | 10.68 | 10.15 | 10.30 | 10.06 |
|  |  |  | SGPC (mg) | 2.49 | 1.58 | 2.04 | 1.91 | 2.16 | 1.93 | 2.00 | 2.04 | 2.04 |
| % Improvement of protein content over check | | | GPC | 28.61 |  | 21.17 | 28.19 | 25.87 | 28.66 | 24.45 | 25.25 | 24.27 |
|  |  |  | SGPC | 35.91 |  | 24.63 | 21.40 | 27.48 | 23.83 | 25.02 | 23.58 | 25.71 |
| Mean Grain yield (kg/ha) | | |  | 2450.00 | 4060.00 | 3890.00 | 3870.00 | 3900.00 | 4310.00 | 4246.00 | 3990.00 | 3880.00 |
| % Improvement of grain yield over check (Naveen) | | |  |  |  | -4.19 | -4.68 | -3.94 | 6.16 | 4.58 | -1.72 | -4.43 |
| Mean Protein yield (kg/ha) | | |  | 265.58 | 321.96 | 386.28 | 417.96 | 425.49 | 460.308 | 430.969 | 410.97 | 390.328 |
| % improvement protein yield over check (Naveen) | | |  |  |  |  | 19.97 | 29.82 | 32.16 | 42.97 | 33.86 | 27.65 |

| Supplementary Table 14. Grain quality of introgression lines and their parents | | | | | | |  |  |  |  |  |  |  |  | | |  |
| --- | --- | --- | --- | --- | --- | --- | --- | --- | --- | --- | --- | --- | --- | --- | --- | --- | --- |
| LINES | Hulling (%) | Milling (%) | HRR (%) | Grain length (mm) | Grain breadth (mm) | L/B ratio | Grain type | ASV | Amylose (%) | CRE (%) | VER |  |  |  |  |  |  |
| ARC-10075 | 77.5 | 69 | 62 | 5.6 | 2.21 | 2.53 | SB | 3 | 21.2 | 67.86 | 3.75 |  |  |  |  |  |  |
| Naveen | 74 | 63.5 | 60 | 4.98 | 1.9 | 2.62 | SB | 3 | 21.53 | 60.64 | 4 |  |  |  |  |  |  |
| PLN-23 | 78 | 70 | 66 | 5.4 | 1.92 | 2.81 | SB | 3 | 20.93 | 81.48 | 3.75 |  |  |  |  |  |  |
| PLN-32 | 77 | 67 | 66 | 5.05 | 1.7 | 2.97 | SB | 3 | 22.02 | 74.26 | 3.75 |  |  |  |  |  |  |
| PLN-37 | 76.5 | 68 | 60 | 5.04 | 1.8 | 2.80 | SB | 3 | 21.53 | 82.54 | 3.75 |  |  |  |  |  |  |
| PLN-98 | 77 | 69 | 67 | 6.22 | 1.78 | 3.49 | LS | 3 | 20.45 | 63.99 | 4.25 |  |  |  |  |  |  |
| PLN-99 | 74.5 | 62.5 | 54 | 5.12 | 1.81 | 2.83 | SB | 3 | 22.5 | 89.45 | 3.75 |  |  |  |  |  |  |
| PLN-100 | 78.5 | 70 | 62 | 5.56 | 1.78 | 3.12 | MS | 3 | 20.52 | 74.46 | 4 |  |  |  |  |  |  |
| PLN-116 | 73.5 | 68 | 64 | 4.98 | 1.9 | 2.62 | SB | 3 | 20.07 | 8.3 | 3.75 | 82.5 | | | 11.48 |  |  |

HRR: head rice recovery, VER: volume expansion ratio, CRE: cooked rice elongation, ASV: alkali spreading value

Supplementary Table 15.Rice germplasm with high GPC in brown rice

| Sl.No. | Varieties with high GPC | GPC (%) |
| --- | --- | --- |
| 1 | Kalinga-III | 12.80 |
| 2 | Bindli | 13.20 |
| 3 | PB140 | 12.80 |
| 4 | Mamihunger | 13.60 |
| 5 | ARC10075 | 12.70 |
| 6 | ARC10063 | 12.50 |
| 7 | PB170 | 12.60 |
| 8 | PB- 84 | 13.05 |
| 9 | PB-312 | 14.10 |

Supplementary Table 16. Embryo and Endosperm gene expression profile data at grain developmental stage from RiceXPro database (RXP_0012) (<http://ricexpro.dna.affrc.go.jp/>)

| **NO** | **DATA SET ID** | **SAMPLE ID** | **SAMPLING DETAILS** | **cRNA QTY** | **GRC#** | **Structure Development Stage** | **Anatomical Entity** |
| --- | --- | --- | --- | --- | --- | --- | --- |
| 1 | RXP_0012 | Embryo_07 DAF | Embryo at 07 days after flowering 1_1 | 1650 | 641 | [PO:0007130](http://www.plantontology.org/amigo/go.cgi?view=details&query=PO:0007130) : sporophyte reproductive stage [PO:0001170](http://www.plantontology.org/amigo/go.cgi?view=details&query=PO:0001170) : seed development stage | [PO:0009009](http://www.plantontology.org/amigo/go.cgi?view=details&query=PO:0009009) : plant embryo |
| 2 | RXP_0012 |  | Embryo at 07 days after flowering 1_2 | 1650 | 642 | [PO:0007130](http://www.plantontology.org/amigo/go.cgi?view=details&query=PO:0007130) : sporophyte reproductive stage [PO:0001170](http://www.plantontology.org/amigo/go.cgi?view=details&query=PO:0001170) : seed development stage | [PO:0009009](http://www.plantontology.org/amigo/go.cgi?view=details&query=PO:0009009) : plant embryo |
| 3 | RXP_0012 |  | Embryo at 07 days after flowering 1_3 | 1650 | 643 | [PO:0007130](http://www.plantontology.org/amigo/go.cgi?view=details&query=PO:0007130) : sporophyte reproductive stage [PO:0001170](http://www.plantontology.org/amigo/go.cgi?view=details&query=PO:0001170) : seed development stage | [PO:0009009](http://www.plantontology.org/amigo/go.cgi?view=details&query=PO:0009009) : plant embryo |
| 4 | RXP_0012 | Embryo_10 DAF | Embryo at 10 days after flowering 1_1 | 1650 | 644 | [PO:0007130](http://www.plantontology.org/amigo/go.cgi?view=details&query=PO:0007130) : sporophyte reproductive stage [PO:0001170](http://www.plantontology.org/amigo/go.cgi?view=details&query=PO:0001170) : seed development stage | [PO:0009009](http://www.plantontology.org/amigo/go.cgi?view=details&query=PO:0009009) : plant embryo |
| 5 | RXP_0012 |  | Embryo at 10 days after flowering 1_3 | 1650 | 595 | [PO:0007130](http://www.plantontology.org/amigo/go.cgi?view=details&query=PO:0007130) : sporophyte reproductive stage [PO:0001170](http://www.plantontology.org/amigo/go.cgi?view=details&query=PO:0001170) : seed development stage | [PO:0009009](http://www.plantontology.org/amigo/go.cgi?view=details&query=PO:0009009) : plant embryo |
| 6 | RXP_0012 |  | Embryo at 10 days after flowering 1_4 | 1650 | 596 | [PO:0007130](http://www.plantontology.org/amigo/go.cgi?view=details&query=PO:0007130) : sporophyte reproductive stage [PO:0001170](http://www.plantontology.org/amigo/go.cgi?view=details&query=PO:0001170) : seed development stage | [PO:0009009](http://www.plantontology.org/amigo/go.cgi?view=details&query=PO:0009009) : plant embryo |
| 7 | RXP_0012 | Embryo_14 DAF | Embryo at 14 days after flowering 1_1 | 1650 | 597 | [PO:0007130](http://www.plantontology.org/amigo/go.cgi?view=details&query=PO:0007130) : sporophyte reproductive stage [PO:0001170](http://www.plantontology.org/amigo/go.cgi?view=details&query=PO:0001170) : seed development stage | [PO:0009009](http://www.plantontology.org/amigo/go.cgi?view=details&query=PO:0009009) : plant embryo |
| 8 | RXP_0012 |  | Embryo at 14 days after flowering 1_2 | 1650 | 598 | [PO:0007130](http://www.plantontology.org/amigo/go.cgi?view=details&query=PO:0007130) : sporophyte reproductive stage [PO:0001170](http://www.plantontology.org/amigo/go.cgi?view=details&query=PO:0001170) : seed development stage | [PO:0009009](http://www.plantontology.org/amigo/go.cgi?view=details&query=PO:0009009) : plant embryo |
| 9 | RXP_0012 |  | Embryo at 14 days after flowering 1_3 | 1650 | 599 | [PO:0007130](http://www.plantontology.org/amigo/go.cgi?view=details&query=PO:0007130) : sporophyte reproductive stage [PO:0001170](http://www.plantontology.org/amigo/go.cgi?view=details&query=PO:0001170) : seed development stage | [PO:0009009](http://www.plantontology.org/amigo/go.cgi?view=details&query=PO:0009009) : plant embryo |
| 10 | RXP_0012 | Embryo_21 DAF | Embryo at 21 days after flowering 1_1 | 1650 | 761 | [PO:0007130](http://www.plantontology.org/amigo/go.cgi?view=details&query=PO:0007130) : sporophyte reproductive stage [PO:0001170](http://www.plantontology.org/amigo/go.cgi?view=details&query=PO:0001170) : seed development stage | [PO:0009009](http://www.plantontology.org/amigo/go.cgi?view=details&query=PO:0009009) : plant embryo |
| 11 | RXP_0012 |  | Embryo at 21 days after flowering 1_2 | 1650 | 762 | [PO:0007130](http://www.plantontology.org/amigo/go.cgi?view=details&query=PO:0007130) : sporophyte reproductive stage [PO:0001170](http://www.plantontology.org/amigo/go.cgi?view=details&query=PO:0001170) : seed development stage | [PO:0009009](http://www.plantontology.org/amigo/go.cgi?view=details&query=PO:0009009) : plant embryo |
| 12 | RXP_0012 |  | Embryo at 21 days after flowering 1_3 | 1650 | 763 | [PO:0007130](http://www.plantontology.org/amigo/go.cgi?view=details&query=PO:0007130) : sporophyte reproductive stage [PO:0001170](http://www.plantontology.org/amigo/go.cgi?view=details&query=PO:0001170) : seed development stage | [PO:0009009](http://www.plantontology.org/amigo/go.cgi?view=details&query=PO:0009009) : plant embryo |
| 13 | RXP_0012 | Embryo_28 DAF | Embryo at 28 days after flowering 1_1 | 1650 | 600 | [PO:0007130](http://www.plantontology.org/amigo/go.cgi?view=details&query=PO:0007130) : sporophyte reproductive stage [PO:0001170](http://www.plantontology.org/amigo/go.cgi?view=details&query=PO:0001170) : seed development stage | [PO:0009009](http://www.plantontology.org/amigo/go.cgi?view=details&query=PO:0009009) : plant embryo |
| 14 | RXP_0012 |  | Embryo at 28 days after flowering 1_2 | 1650 | 601 | [PO:0007130](http://www.plantontology.org/amigo/go.cgi?view=details&query=PO:0007130) : sporophyte reproductive stage [PO:0001170](http://www.plantontology.org/amigo/go.cgi?view=details&query=PO:0001170) : seed development stage | [PO:0009009](http://www.plantontology.org/amigo/go.cgi?view=details&query=PO:0009009) : plant embryo |
| 15 | RXP_0012 |  | Embryo at 28 days after flowering 1_3 | 1650 | 602 | [PO:0007130](http://www.plantontology.org/amigo/go.cgi?view=details&query=PO:0007130) : sporophyte reproductive stage [PO:0001170](http://www.plantontology.org/amigo/go.cgi?view=details&query=PO:0001170) : seed development stage | [PO:0009009](http://www.plantontology.org/amigo/go.cgi?view=details&query=PO:0009009) : plant embryo |
| 16 | RXP_0012 | Embryo_42 DAF | Embryo at 42 days after flowering 1_1 | 1650 | 603 | [PO:0007130](http://www.plantontology.org/amigo/go.cgi?view=details&query=PO:0007130) : sporophyte reproductive stage [PO:0001170](http://www.plantontology.org/amigo/go.cgi?view=details&query=PO:0001170) : seed development stage | [PO:0009009](http://www.plantontology.org/amigo/go.cgi?view=details&query=PO:0009009) : plant embryo |
| 17 | RXP_0012 |  | Embryo at 42 days after flowering 1_2 | 1650 | 604 | [PO:0007130](http://www.plantontology.org/amigo/go.cgi?view=details&query=PO:0007130) : sporophyte reproductive stage [PO:0001170](http://www.plantontology.org/amigo/go.cgi?view=details&query=PO:0001170) : seed development stage | [PO:0009009](http://www.plantontology.org/amigo/go.cgi?view=details&query=PO:0009009) : plant embryo |
| 18 | RXP_0012 |  | Embryo at 42 days after flowering 1_3 | 1650 | 605 | [PO:0007130](http://www.plantontology.org/amigo/go.cgi?view=details&query=PO:0007130) : sporophyte reproductive stage [PO:0001170](http://www.plantontology.org/amigo/go.cgi?view=details&query=PO:0001170) : seed development stage | [PO:0009009](http://www.plantontology.org/amigo/go.cgi?view=details&query=PO:0009009) : plant embryo |
| 19 | RXP_0012 | Endosperm_07 DAF | Endosperm at 07 days after flowering 1_1 | 1650 | 606 | [PO:0007130](http://www.plantontology.org/amigo/go.cgi?view=details&query=PO:0007130) : sporophyte reproductive stage [PO:0001170](http://www.plantontology.org/amigo/go.cgi?view=details&query=PO:0001170) : seed development stage | [PO:0009089](http://www.plantontology.org/amigo/go.cgi?view=details&query=PO:0009089) : endosperm |
| 20 | RXP_0012 |  | Endosperm at 07 days after flowering 1_2 | 1650 | 607 | [PO:0007130](http://www.plantontology.org/amigo/go.cgi?view=details&query=PO:0007130) : sporophyte reproductive stage [PO:0001170](http://www.plantontology.org/amigo/go.cgi?view=details&query=PO:0001170) : seed development stage | [PO:0009089](http://www.plantontology.org/amigo/go.cgi?view=details&query=PO:0009089) : endosperm |
| 21 | RXP_0012 |  | Endosperm at 07 days after flowering 1_3 | 1650 | 608 | [PO:0007130](http://www.plantontology.org/amigo/go.cgi?view=details&query=PO:0007130) : sporophyte reproductive stage [PO:0001170](http://www.plantontology.org/amigo/go.cgi?view=details&query=PO:0001170) : seed development stage | [PO:0009089](http://www.plantontology.org/amigo/go.cgi?view=details&query=PO:0009089) : endosperm |
| 22 | RXP_0012 | Endosperm_10 DAF | Endosperm at 10 days after flowering 1_1 | 1650 | 609 | [PO:0007130](http://www.plantontology.org/amigo/go.cgi?view=details&query=PO:0007130) : sporophyte reproductive stage [PO:0001170](http://www.plantontology.org/amigo/go.cgi?view=details&query=PO:0001170) : seed development stage | [PO:0009089](http://www.plantontology.org/amigo/go.cgi?view=details&query=PO:0009089) : endosperm |
| 23 | RXP_0012 |  | Endosperm at 10 days after flowering 1_2 | 1650 | 610 | [PO:0007130](http://www.plantontology.org/amigo/go.cgi?view=details&query=PO:0007130) : sporophyte reproductive stage [PO:0001170](http://www.plantontology.org/amigo/go.cgi?view=details&query=PO:0001170) : seed development stage | [PO:0009089](http://www.plantontology.org/amigo/go.cgi?view=details&query=PO:0009089) : endosperm |
| 24 | RXP_0012 |  | Endosperm at 10 days after flowering 1_3 | 1650 | 611 | [PO:0007130](http://www.plantontology.org/amigo/go.cgi?view=details&query=PO:0007130) : sporophyte reproductive stage [PO:0001170](http://www.plantontology.org/amigo/go.cgi?view=details&query=PO:0001170) : seed development stage | [PO:0009089](http://www.plantontology.org/amigo/go.cgi?view=details&query=PO:0009089) : endosperm |
| 25 | RXP_0012 | Endosperm_14 DAF | Endosperm at 14 days after flowering 1_1 | 1650 | 612 | [PO:0007130](http://www.plantontology.org/amigo/go.cgi?view=details&query=PO:0007130) : sporophyte reproductive stage [PO:0001170](http://www.plantontology.org/amigo/go.cgi?view=details&query=PO:0001170) : seed development stage | [PO:0009089](http://www.plantontology.org/amigo/go.cgi?view=details&query=PO:0009089) : endosperm |
| 26 | RXP_0012 |  | Endosperm at 14 days after flowering 1_2 | 1650 | 613 | [PO:0007130](http://www.plantontology.org/amigo/go.cgi?view=details&query=PO:0007130) : sporophyte reproductive stage [PO:0001170](http://www.plantontology.org/amigo/go.cgi?view=details&query=PO:0001170) : seed development stage | [PO:0009089](http://www.plantontology.org/amigo/go.cgi?view=details&query=PO:0009089) : endosperm |
| 27 | RXP_0012 |  | Endosperm at 14 days after flowering 1_3 | 1650 | 614 | [PO:0007130](http://www.plantontology.org/amigo/go.cgi?view=details&query=PO:0007130) : sporophyte reproductive stage [PO:0001170](http://www.plantontology.org/amigo/go.cgi?view=details&query=PO:0001170) : seed development stage | [PO:0009089](http://www.plantontology.org/amigo/go.cgi?view=details&query=PO:0009089) : endosperm |
| 28 | RXP_0012 | Endosperm_21 DAF | Endosperm at 21 days after flowering 1_1 | 1650 | 764 | [PO:0007130](http://www.plantontology.org/amigo/go.cgi?view=details&query=PO:0007130) : sporophyte reproductive stage [PO:0001170](http://www.plantontology.org/amigo/go.cgi?view=details&query=PO:0001170) : seed development stage | [PO:0009089](http://www.plantontology.org/amigo/go.cgi?view=details&query=PO:0009089) : endosperm |
| 29 | RXP_0012 |  | Endosperm at 21 days after flowering 1_2 | 1650 | 765 | [PO:0007130](http://www.plantontology.org/amigo/go.cgi?view=details&query=PO:0007130) : sporophyte reproductive stage [PO:0001170](http://www.plantontology.org/amigo/go.cgi?view=details&query=PO:0001170) : seed development stage | [PO:0009089](http://www.plantontology.org/amigo/go.cgi?view=details&query=PO:0009089) : endosperm |
| 30 | RXP_0012 |  | Endosperm at 21 days after flowering 1_3 | 1650 | 766 | [PO:0007130](http://www.plantontology.org/amigo/go.cgi?view=details&query=PO:0007130) : sporophyte reproductive stage [PO:0001170](http://www.plantontology.org/amigo/go.cgi?view=details&query=PO:0001170) : seed development stage | [PO:0009089](http://www.plantontology.org/amigo/go.cgi?view=details&query=PO:0009089) : endosperm |
| 31 | RXP_0012 | Endosperm_28 DAF | Endosperm at 28 days after flowering 1_1 | 1650 | 615 | [PO:0007130](http://www.plantontology.org/amigo/go.cgi?view=details&query=PO:0007130) : sporophyte reproductive stage [PO:0001170](http://www.plantontology.org/amigo/go.cgi?view=details&query=PO:0001170) : seed development stage | [PO:0009089](http://www.plantontology.org/amigo/go.cgi?view=details&query=PO:0009089) : endosperm |
| 32 | RXP_0012 |  | Endosperm at 28 days after flowering 1_2 | 1650 | 616 | [PO:0007130](http://www.plantontology.org/amigo/go.cgi?view=details&query=PO:0007130) : sporophyte reproductive stage [PO:0001170](http://www.plantontology.org/amigo/go.cgi?view=details&query=PO:0001170) : seed development stage | [PO:0009089](http://www.plantontology.org/amigo/go.cgi?view=details&query=PO:0009089) : endosperm |
| 33 | RXP_0012 |  | Endosperm at 28 days after flowering 1_3 | 1650 | 617 | [PO:0007130](http://www.plantontology.org/amigo/go.cgi?view=details&query=PO:0007130) : sporophyte reproductive stage [PO:0001170](http://www.plantontology.org/amigo/go.cgi?view=details&query=PO:0001170) : seed development stage | [PO:0009089](http://www.plantontology.org/amigo/go.cgi?view=details&query=PO:0009089) : endosperm |
| 34 | RXP_0012 | Endosperm_42 DAF | Endosperm at 42 days after flowering 1_1 | 1650 | 618 | [PO:0007130](http://www.plantontology.org/amigo/go.cgi?view=details&query=PO:0007130) : sporophyte reproductive stage [PO:0001170](http://www.plantontology.org/amigo/go.cgi?view=details&query=PO:0001170) : seed development stage | [PO:0009089](http://www.plantontology.org/amigo/go.cgi?view=details&query=PO:0009089) : endosperm |
| 35 | RXP_0012 |  | Endosperm at 42 days after flowering 1_2 | 1650 | 619 | [PO:0007130](http://www.plantontology.org/amigo/go.cgi?view=details&query=PO:0007130) : sporophyte reproductive stage [PO:0001170](http://www.plantontology.org/amigo/go.cgi?view=details&query=PO:0001170) : seed development stage | [PO:0009089](http://www.plantontology.org/amigo/go.cgi?view=details&query=PO:0009089) : endosperm |
| 36 | RXP_0012 |  | Endosperm at 42 days after flowering 1_3 | 1650 | 620 | [PO:0007130](http://www.plantontology.org/amigo/go.cgi?view=details&query=PO:0007130) : sporophyte reproductive stage [PO:0001170](http://www.plantontology.org/amigo/go.cgi?view=details&query=PO:0001170) : seed development stage | [PO:0009089](http://www.plantontology.org/amigo/go.cgi?view=details&query=PO:0009089) : endosperm |

| **Table Header** | |
| --- | --- |
| **DATA SET ID** | Data set identifier |
| **SAMPLE ID** | Expression profile identifier |
| **SAMPLING DETAILS** | Date/time of sampling, sample no., actual sample size, sample size relative to another organ, other information |
| **GROWTH STAGE** | Stage or phase of growth based on specific events such as transplanting, flowering etc. |
| **cRNA QTY** | Amount of cRNA (ng) used for hybridization |
| **GRC#** | Experiment / expression analysis identification number |
| **Structure Development Stage** | [Plant Ontology](http://www.plantontology.org/) |
| **Anatomical Entity** | [Plant Ontology](http://www.plantontology.org/) |

Supplementary Figure 1. High-quality SNPs showing well-separated clusters of homozygous alleles


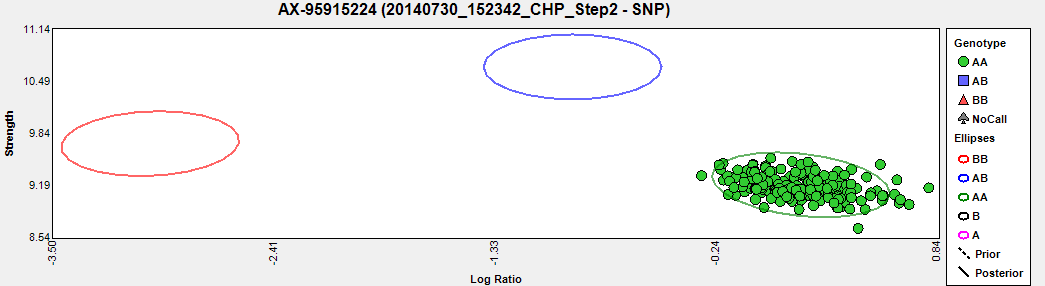


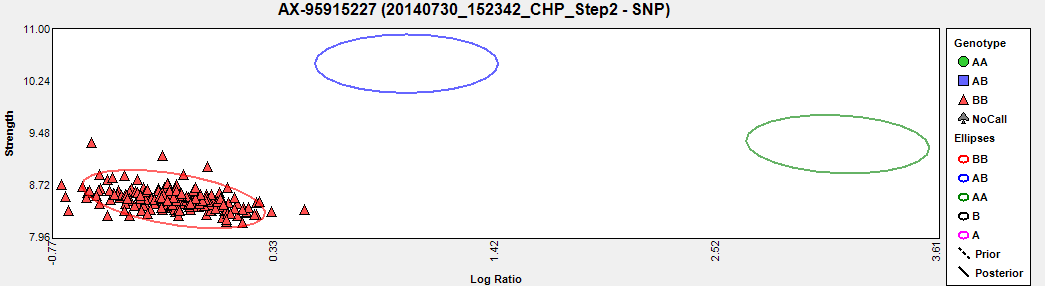


Supplementary Figure 2. Distribution of grain protein content estimated through NIR spectrophotometer of NILs (BC3F5) derived from ARC10075/Naveen cross in *kharif season* 2014 (*Env.* 3)


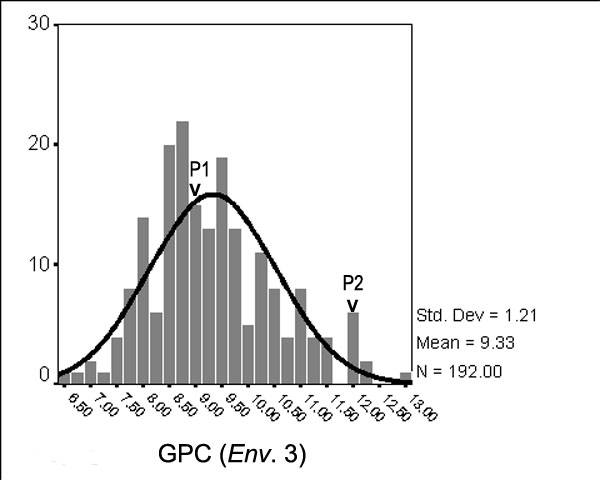


Supplementary Fig. 3. Box plot view of expression profiles of selected genes located inside QTLs for grain protein content at different plant parts based on Rice expression database (RED) from [IC4R website (http://ic4r.org)](file:///C:\Users\DELL\AppData\Local\Temp\IC4R%20website%20(http:\ic4r.org))
